# Automated Ranking of Soybean Plots from Close-Range RGB Video via Depth Filtering and Point-Based Counting

**DOI:** 10.3390/s26196171

**Published:** 2026-09-29

**Authors:** Nikita Teterin, Sergey Slastnikov, Petr Rybakov, Maksim Groshev

**Affiliations:** Tikhonov Moscow Institute of Electronics and Mathematics, HSE University, 20 Myasnitskaya Street, Moscow 101000, Russia; cv.ne.teterin@gmail.com (N.T.); sslastnikov@hse.ru (S.S.); prybakov@hse.ru (P.R.)

**Keywords:** computer vision, deep learning, agricultural automation, zero-shot segmentation, depth estimation, object detection, keypoint detection, soybean phenotyping

## Abstract

Manual assessment of soybean yield components, such as pod number, is laborious, time-consuming, and subjective. Existing computer-vision approaches based on object detection or instance segmentation perform poorly on close-range RGB imagery of soybean canopies due to severe occlusions, ambiguous plant boundaries, and the high cost of bounding-box annotation. To address these challenges, we propose a video-based ranking pipeline that avoids explicit per-plant detection and instead aggregates global frame-level features derived from monocular depth estimation, semantic plant segmentation, and pod keypoint counting. Depth maps from Depth Anything 3 are used to suppress background clutter, while zero-shot segmentation with SAM3 (distilled into a lightweight SegFormer) provides plant-area signals and a point-based counting network (P2PNet) estimates pod counts. We evaluate the method on a controlled dataset comprising 11 ranks with multiple indoor and outdoor scenes. The proposed pipeline provides a practical, cost-effective solution for automated bed-quality scoring, although it currently depends on fixed camera geometry and does not yet associate pods with individual plants.

## 1. Introduction

Computer Vision (CV) applications in agriculture span a vast range of problems, including crop and field monitoring, yield estimation and livestock monitoring. This work focuses on plant phenotyping automation, specifically soybean phenotyping in outdoor conditions using RGB imagery. Recent agronomic studies confirm that pod count is a primary yield determinant. Humtsoe et al. (2017) [1] conducted a comprehensive correlation and path analysis of twelve soybean genotypes. They found that the number of pods per plant has the highest positive direct effect on seed yield at the genotypic level, outperforming traits such as plant height and branch number. Moreover, pod count exhibited strong positive correlation with seed yield per plant and demonstrated high heritability. This makes pod count a reliable selection criterion in breeding programs. This agronomic relevance, combined with the visual saliency of pods in close-range RGB imagery, motivates automated pod counting as a biologically meaningful and technically tractable proxy for plant quality assessment. Manual counting of pods and seeds is labor-intensive, time-consuming and error-prone. Thus, developing tools to automate this process is an important task. The main challenge addressed in this work is that standard object detection and segmentation methods fail on close-range soybean imagery due to dense canopy occlusion, ambiguous plant boundaries, and the high cost of manual bounding-box annotation (Figure 1). Instead of attempting per-plant localization, we reformulate the task as video-level bed ranking using aggregate frame-level features.

Recent studies are shifting focus from controlled indoor laboratory environments to open, outdoor spaces such as fields to shorten the gap between ideal and real-world conditions and address genotype interaction with the specific environment. While autonomous driving requires detecting distinct, rigid objects (e.g., cars, pedestrians) on a plane, plant phenotyping deals with non-rigid, visually homogenic, fractal structures that change over time. Other problems include dynamic scale and lighting of plants, dense clutter (e.g., between leaves and other organs), strong background occlusions, significant influence of camera position and distance to the object of interest. For most agricultural tasks, targets are small and dense, which makes human labeling a very time-expensive process. In our study, annotation of a single image with soybean pod keypoints takes around 6 min, which makes it a major bottleneck for prototyping. However, fully automatic annotation remains inapplicable in this domain. No existing model—including zero-shot foundation models such as SAM3 or Grounding DINO—can produce sufficiently accurate and stable labels for close-range soybean imagery, as evidenced by their tendency to over-segment canopies or miss plants entirely. The annotation process is discussed in more detail in Section 3.3.

The challenges outlined above motivate a careful survey of the state-of-the-art methods in visual recognition. In Section 2, we trace the evolution of 2D object detection from single-stage convolutional regressors to end-to-end Transformer-based detectors, examining how architectural innovations address the dense, small-object regimes characteristic of plant phenotyping. We further review Visual Foundation Models—self-supervised and open-vocabulary systems whose rich, transferable representations offer a promising path to reducing dependence on costly hand-labeled agricultural data. Point-based and counting frameworks, which replace bounding-box annotations with cheaper keypoint supervision, are surveyed as a direct response to the labeling bottleneck identified above. Despite the rapid progress in visual foundation models and general-purpose detectors, their direct application to agricultural phenotyping remains problematic. Plant canopies exhibit fractal, non-rigid structures with severe mutual occlusion and dynamic scale variations. They violate the assumptions underlying typical detection benchmarks. Moreover, manual annotation is prohibitively time-consuming, and whole-plant bounding boxes are inherently ambiguous due to diffuse plant boundaries. These challenges motivate our core hypothesis: instead of per-plant detection, entire plots can be ranked using aggregate frame-level signals that are more robust to visual clutter. The main contributions of this work are:

1. Systematic empirical evidence that state-of-the-art object detectors (YOLO, RT-DETR) and zero-shot foundation models (Grounding DINO, SAM3) are unreliable for close-range soybean imagery, challenging the assumption that general-purpose vision models can be directly applied to agricultural phenotyping.
2. A novel video-level ranking pipeline that integrates monocular depth estimation, semantic segmentation, and point-based pod counting to extract aggregate quality signals—plant area and pod count—without requiring per-plant detection or costly bounding-box annotations.
3. A controlled ranking dataset with natural monotonic order (11 ranks, 22 scenes, indoor and outdoor) and an evaluation protocol. Using this dataset, we show that mean pod count consistently outperforms area-based features and median aggregation, achieving Kendall τ>0.91 (indoor) and >0.83 (outdoor). This demonstrates that automated pod counting is a reliable, agronomically relevant proxy for bed-level quality assessment, with potential to accelerate soybean breeding programs.

First, a survey of methods and practices for object detection and analysis is discussed throughout Section 2. Second, Section 3 outlines the key constraints of the problem considered in this work, describes a series of experiments with zero-shot methods, and formulates plant analysis as a bounding-box detection task. Finally, Section 4 presents the proposed ranking method, along with data collection, evaluation procedures and results.

## 2. Related Works

The agricultural domain in computer vision introduces a unique combination of challenges. Plants can be considered as semi-rigid, fractal-like structures that can look vastly different depending on the camera angle. Another challenge is the visual homogeneity of clustered plants (i.e., trees in a forest or bushes on a bed), which makes features and boundaries of singular plants hardly distinguishable. Moreover, when agricultural images are captured in a natural environment, all challenges related to weather and lighting conditions are applied. Finally, the variety of agricultural plants and narrowness of the domain make usable data rather scarce. Object detection has evolved rapidly. The YOLO family [2,3] popularized single-stage, real-time detection by framing the task as a regression problem over a grid of anchor boxes; modern versions (YOLOv8, YOLOv11) use anchor-free heads and decoupled classification-regression branches. Transformer-based detectors such as DETR [4] reformulated detection as a set-prediction problem, eliminating the need for non-maximum suppression and hand-crafted anchors. Variants like Deformable DETR [5] and DINO detector [6] addressed the slow convergence and quadratic complexity of vanilla DETR through deformable attention and contrastive query denoising. These models achieve state-of-the-art results on general benchmarks (COCO, ImageNet) but are typically trained on large, diverse datasets of everyday objects. When fine-tuned to agricultural imagery, they often overfit or produce unstable predictions due to the domain shift and the intrinsic ambiguity of plant boundaries.

Visual foundation models (VFMs) promise zero-shot generalization by learning rich representations from massive, weakly supervised data. Self-supervised models like DINO detector [7] and DINO detector v2 [8] produce semantically meaningful features without labels, enabling linear probes for segmentation and depth estimation. Contrastive language–image pre-training (CLIP) [9] aligns visual and textual embeddings, allowing zero-shot classification via text prompts. The Segment Anything Model (SAM) [10] introduced promptable segmentation: given a point, box, or text, it can generate object masks for nearly any image, trained on the large-scale SA-1B dataset. SAM2 [11] extended this to video, and SAM3 [12] added support for conceptual prompts (e.g., “soybean plant”). These models have been successfully integrated into pipelines like Grounded SAM [13], combining open-vocabulary detection (Grounding DINO DINO detector [14]) with SAM for zero-shot instance segmentation. Despite their power, VFMs are not a panacea for agricultural applications. Several recent studies have explored their use in phenotyping: Oehme et al. [15] reported a substantial reduction in annotation time (from 9.7 s to 2.1 s per mask) using SAM-assisted crop annotation. Ghose et al. [16] applied a similar hybrid approach to strawberry detection and segmentation. However, for dense, highly occluded targets such as soybean pods, we observed that zero-shot detection (Grounding DINO, OWL-ViT [17]) and segmentation (SAM3) are unstable. They often miss entire plants or over-segment canopies, producing masks that encompass more than 80% of the image area. These failures motivated us to pursue an alternative strategy based on point-level supervision rather than bounding boxes or dense masks.

When objects are small, numerous, and heavily overlapping, bounding-box annotation becomes both costly and ambiguous. In crowd counting, a widely adopted solution is to predict density maps: each ground-truth point is convolved with a Gaussian kernel, yielding a continuous spatial distribution whose integral equals the count. Models like MCNN [18] and CSRNet [19] use multi-column or dilated convolutions to handle scale variation and achieve good counting accuracy. However, density maps provide only a smooth probability surface, not individual object locations, which prevents per-object analysis (e.g., associating pods with stems).

Point-to-Point Network (P2PNet) [20] reframes counting as direct set prediction: given an image, it outputs a set of point coordinates, one per object, using a VGG-16 backbone and a Hungarian matching loss similar to DETR. This approach yields both the count and the spatial locations of each object, with only point-level annotations required—which are far cheaper than bounding boxes. P2PNet has been successfully applied to agricultural counting, e.g., for soybean seeds [21] and rice grains [22], albeit on high-resolution images with minimal occlusions.

Our setup differs substantially: we use a wide-angle camera at close range (15–30 cm), capturing entire soybean canopies with severe perspective distortion and overlapping leaves. In order to address challenges of the domain, we incorporate a depth-filtering preprocessing step (Depth Anything 3 [23]) to perform depth filtering, then we apply P2PNet for pod detection. To improve stability, we train P2PNet and use Slicing-Aided Hyper-Inference (SAHI) [24] to process high-resolution images in overlapping patches. Our experiments show that this pipeline provides reliable pod-count signals that correlate well with manual ranking.

The choice of pod count as a quality indicator is not merely technical but is grounded in agronomic research. Humtsoe et al. [1] found that the number of pods per plant has a highly positive direct effect on seed yield at the genotypic level, making it one of the primary selection criteria in breeding programs. Automating pod counting thus directly addresses a practical need, complementing other yield-related traits such as seed weight or biomass.

In summary, the surveyed literature reveals a tension: while general-purpose object detectors and foundation models have achieved remarkable success on common benchmarks, they are ill-suited for close-range, occluded agricultural scenes. Supervised fine-tuning of YOLO or DETR is hampered by ambiguous bounding-box annotations and rapid overfitting. Zero-shot approaches (SAM3, Grounding DINO) are occasionally useful for annotation acceleration but lack the stability required for automated ranking. By contrast, point-based counting offers a practical middle ground: cheap point annotations, explicit instance localization, and direct compatibility with downstream tasks (e.g., pod-stem association). Our proposed pipeline builds on these insights, combining depth filtering, segmentation, and point counting to rank soybean plots without ever detecting individual plants.

## 3. Materials and Methods

### 3.1. Constraints and Data Collection

Soy phenotyping tasks introduce various challenges such as lighting and weather conditions, camera angle and cluttered scene. Since we have chosen pod count as the main feature to evaluate yield, we had to come up with a camera placement that will allow a clear side view of plants. In this study, “outdoor conditions” refer to recordings made on open-air test beds where soybean plants were grown in containers or small plots, not in commercial fields. This setup allows controlled variation of plant quality while still capturing natural lighting and background variations. For yield-related traits such as pod count and seed filling, a lateral camera view is more informative than a top-down perspective: it captures vertical distribution of pods along the stem and provides a clearer visual signature of fruiting nodes.

Among the possible acquisition platforms—handheld camera, drone, or ground rover—we selected a manually operated trolley with a fixed wide-angle camera. It ensures consistent viewpoint and distance while allowing close-range (15–30 cm) side-view recordings that are both repeatable and easy to operate without specialized training. The prototype of this trolley was developed separately (Figure 2); the location of the camera is highlighted with a red rectangle in Figure 2a.

Another practical constraint for the proposed system is crop bed dimensions. In our particular case, crop beds are planted using machinery that creates grids of 5 parallel 1 m long rows with 10 plants in each with a 50 cm intra-row distance. Such narrow row placement leaves little space for a camera, with the possible distance to object ranging between 20 and 40 cm. To address these constraints, we selected a camera equipped with an Onsemi AR0234CS 1/2.6-inch, 2.3-Megapixel global shutter sensor (Onsemi, Scottsdale, AZ, USA), paired with a 1.8 mm focal length lens. This camera was sufficient to capture the full height of the test crops when the placed at 25 cm from the test bed. No fisheye camera calibration was used since it would significantly limit viewing angle, which is unacceptable for this use case. As further experiments show, distortions from the short focal range lens were not critical for algorithms effectiveness.

As a remark, during indoor experiments, a small rover bot was tested with the same camera (Figure 3). It was not used in the proposed pipeline, although the general approach described by this work can be migrated to a different platform such as that bot.

Samples obtained during prior research in outdoor field conditions (Figure 4) demonstrate several issues commonly seen in agricultural applications listed earlier: high background noise from distant plants, vague instance borders due to canopy occlusion, complemented by perspective distortions near frame borders. These challenges and constraints shaped the design decisions made in this work.

### 3.2. Zero-Shot Model Performance

Several kinds of zero-shot models were tested on our domain, the first being depth estimation models from the Depth Anything family [23,25]. One key idea is that depth filtering may improve the performance of downstream detection and segmentation models by removing major noise. Another important observation is that models trained on such data achieve background invariance, i.e., they can be trained on indoor imagery using strong color augmentations and then be applied to outdoor conditions. Depth Anything 2 [25] achieved good performance on our domain, successfully separating foreground plants from background with high precision. Depth Anything 3 [23], released in November 2025, further improves depth map quality and model architecture, which now allows multi-view geometry reconstruction via camera parameter estimation (intrinsic and extrinsic matrices). There are several ways to implement background subtraction, with two of the most straightforward being constant threshold cutoff or adaptive (e.g., percentile-based) cutoff. The original dataset of field videos did not have stable reference points for a fixed threshold. Depth distribution examples with varying depth distributions are presented in Figure 5. The percentile-based method is generally more robust, but it is still error-prone. In the conducted experiments, a 55th-percentile cutoff provided the most stable foreground separation across the evaluated scenes. This idea of background subtraction is further justified by the target deployment platform. Images collected from a trolley provide highly consistent reference points on the metal frame, which can be used to compute fixed cutoff threshold, which turned out to work quite well and became the first preprocessing step in the proposed CV pipeline (Figure 6).

Zero-shot detectors can theoretically solve complex vision and multi-modal tasks with minimal effort. They are, however, general task solvers and often fail on domain-specific problems. Plant detection proved to be a task with high ambiguity under the given imaging conditions for recent models like Grounding DINO detector [14] and OWL-ViT [17]. Experiments show that plants are detected for simple scenes with a single plant in the center but fail on more complex scenarios, as presented in Figure 7. Few-shot adaptation for these models was attempted but did not yield substantial improvement, although it might improve their performance or at least make such detectors viable for pseudo-label generation for manual review.

Expanding on model-assisted annotation, the Segment Anything Model family was also tested. These are commonly used to accelerate the annotation process, when a person draws bounding boxes or points that act as visual prompts for the model. The same idea is implemented in Grounded SAM [13], which uses open vocabulary detector outputs as visual prompts for SAM. This approach is widely adopted but still requires a robust object detection model. As argued earlier, zero-shot detectors were insufficient for the target imagery; thus, visual prompting was of limited practical utility in this setting. An interactive setup with LabelStudio [26] was tested with the SAM2 model backend for interactive annotation (Figure 8). It has good potential for assisted mask generation for small and medium objects that are straightforward to draw a bounding box around (e.g., individual visible soybean pods). However, SAM2 still fails on complex object boundaries and struggles with canopy occlusion of soybean plants. In order to segment such shapes, more visual prompts are required, which defeats the original goal.

A major update in the SAM family happened in [12], which finally bridged the gap between great mask quality and text modality via conceptual prompts. In practice, this allowed robust zero-shot mask generation using text prompts only. SAM3 exhibited occasional instability on the collected data, as shown in Figure 9. Still, there are two main ways to employ SAM3 to our problem: zero-shot segmentation and pseudo-label generation for object detection. The former achieves acceptable performance but requires expensive forward passes through a large model and is still unstable. The latter requires human supervision but can substantially increase the speed of bounding-box annotation (since any mask can be converted to a bounding box). Both approaches were tested, yet a thorough review revealed that generated boxes are usually too large and tend to contain more than 80% of the image area, as shown in Figure 9.

In order to improve zero-shot mask quality, one idea involved combining SAM3 with depth map estimation and background subtraction, which was infeasible for the original soybean field dataset due to inconsistent plant distance and lack of stable reference points across frames. A different dataset was required for further iteration on this method.

### 3.3. Object Detection

This subsection evaluates whether whole-plant detection is a suitable formulation for the collected wide-angle soybean imagery. YOLOv11 and RT-DETR are compared under RGB and depth-filtered scenes, and both detection metrics and qualitative failure modes are analyzed. The results suggest that the models learn coarse plant localization but fail to produce stable and fine-grained boxes, reflected by the gap between mAP@50 and mAP@50-95. Direct pod detection and counting with P2PNet is therefore evaluated as a reasonable substitute for SAM3 in the ranking pipeline. The subsequent experiments reformulate the task as video-level ranking using aggregate plant area and pod-count signals.

The cornerstone of supervised detection training is the volume of available high-quality labeled data. For this research, around 5000 training and 800 validation frames were manually labeled with bounding boxes with a single plant class. Annotations are produced with Computer Vision Annotation Tool (CVAT). It provides convenient keyboard shortcuts for most common operations and useful features like box interpolation across frames, which substantially accelerate annotation of videos since objects move predictably during camera rolls. As experiments show (Table 1), our manual box labels actually make model training unstable, and performance metrics plateau at around 0.75 mAP@50 and 0.4 mAP@50-95. These values signify that models successfully learn a broad notion of soybean plants (good mAP@50) but fail to produce fine-grained boxes (low mAP@50-95), further supported by inspection of model error visualizations. There are two primary reasons for this:

1. First, all plants moving in the video enter and exit the frame at some point. One key problem is to decide whether a currently visible part of the plant (e.g., top of a branch or several branches with leaves) should be considered a valid box target. Since initially, a single video was labeled for detection, many such edge cases appeared and introduced ambiguity, e.g., for two frames that are visually very similar, only one gets a box.
2. Another important issue is differentiation between a single large plant and multiple smaller ones. As a large blob of multiple plants is entering the scene, it is ambiguous when it should stop being considered a single plant and split into two boxes instead. With a wide angle camera, plant appearance changes drastically during the recording, capturing them from the front and the sides. For fractal structures such as plants, this renders strict bounding-box annotations not of much use due to ambiguity and large box overlaps, in contrast to masks, which handle overlaps and perspective distortions. If one wanted to compute per-plant metrics such as yield, a more robust association algorithm is required.

The results obtained in the detection model fine-tuning experiments are presented in Table 1. RT-Detection Transformer (DETR) displayed better performance than YOLOv11 because it has a better backbone, and more aggressive augmentations (color jitter, random Gaussian noise, random brightness and contrast augmentations, contrast-limited adaptive histogram equalization) aided generalization. Unexpectedly, background subtraction using depth maps had a minimal effect on the metrics. All experiments display overfit in less than 10 epochs as validation metrics stop improving. All of these facts then indicate that whole-plant bounding-box detection is not a stable formulation for this dataset. A larger and more consistently annotated detection dataset would likely improve model performance. However, the annotation cost and persistent boundary ambiguity make this formulation poorly aligned with the goals of this work. Since both zero-shot inference and supervised whole-plant detection remained unreliable under the target imaging conditions, this work adopts an alternative formulation based on aggregate video-level ranking signals.

### 3.4. Pod Counting with P2PNet

Point-to-Point Network (P2PNet) [20] model directly predicts point sets in images. It was originally developed for crowd counting with a single class, but it is easy to extend for multi-class keypoint detection. This model has already been successfully applied to soybean and grain phenotyping [21,22,27], with two major distinctions from this work:

1. First, ref. [21] utilizes high-resolution images (2399×1599 and 7728×9228), which appear to be taken with a high-end reflex camera (Figure 10). Plants are clearly visible and separable in the foreground, and soybean pods appear large and thus bean counting is achievable directly, which drastically differs from our setup.
2. Second, ref. [27] performs offline processing with heavy models, while we are targeting online on-device inference.

In pursuit of stability, three classes are used for P2PNet training as opposed to one: soybean pod, stem and metal frame keypoints at fixed locations. Inference examples can be found in Figure 11. Roughly 100 training and 10 validation frames are labeled manually.

EasyP2PNet employs a technique similar to Slicing-Aided Hyper-Inference (SAHI) [24], which slices the original 1920×1200 image into smaller chunks (256×256, 384×384 and 512×512 sizes were tested) and processes each chunk separately. Chunk processing can be batched, although experiments indicate that VGG-16 [28] used for feature extraction in P2PNet actually saturates GPU and performance plateaus near the theoretical FLOPS limit, so increasing batch size further would only increase memory consumption without benefiting performance. A single 1920×1200 image can be split into 40 256×256 patches, which can be processed with batch size of 8 at 5–8 FPS on NVIDIA RTX 5080 using 1.7 G of video memory. SAHI is also used during training: 100 large images are split into overlapping 512×512 patches with an overlap ratio of 0.5, resulting in 3150 training samples. During training, randomly augmented 256×256 crops from 512×512 patches are used as model inputs.

P2PNet is trained from ImageNet VGG-16 checkpoint for 20 epochs with batch size of 32 and reaches MAE of 2.78 and MSE of 3.64 per patch (Table 2). MAE is further amplified since there are 40 patches per image, yet pod counting still proved to be a robust proxy for plant quality ranking.

One notable issue encountered during the experiments is the clustering algorithm employed by the EasyP2PNet implementation: it uses k-means to suppress duplicate predictions. Deduplication radius is an important hyperparameter that depends on spatial resolution and patch size. In a field scenario with camera platform movement, varying target distances cause perspective scale changes: a fixed pixel radius risks leaving duplicate points on large foreground pods while merging correct distinct detections on small distant pods. Incorporating dynamic, depth-aware deduplication radii remains an important direction for robust field deployment.

One specific property of this point-based framework that cannot be easily implemented with segmentation masks is that detected pod points can be spatially associated with plant stem points, which introduces plant-level features. This can be easily done using a greedy stem association method (i.e., each pod point is associated with the $L_{2}$-nearest detected stem point) or the more complex *k*-lines algorithm. This, however, requires a robust stem keypoint detection algorithm. P2PNet was found to not yet be robust enough for this task, presumably due to class imbalance as there are at most about 10 stems per frame and easily more than 100 pods. In our implementation, the classification loss uses standard cross-entropy with uniform weights ($\omega_{c}=1$) across all foreground classes and a reduced coefficient ($\omega_{0}=0.01$) for the background class. To resolve this imbalance, several loss function modifications can be explored:

1. Inverse class frequency weighting: assigning explicit class weights $\omega_{c}\propto 1/N_{c}$ in the cross-entropy term to scale gradient updates for scarce stem points.
2. Replacing classic cross-entropy loss with Focal loss in both classification loss and Hungarian matching cost to dynamically down-weight easy pod and background samples and prioritize ambiguous stem keypoints.

Still, building a robust stem association framework on top of P2PNet detections is a promising research direction.

### 3.5. Pipeline Design

Two versions of our proposed pipeline for soybean plots scoring are presented in Figure 12. It combines engineering heuristics with modern zero-shot feature extraction. First, a depth map is extracted from the source RGB image. Background is then removed using knowledge of metal frame distance, which stays relatively constant across scenes. This step reduces background noise and allows the proposed method to achieve scene invariance across indoor and outdoor scenes since only plants captured by the frame are kept visible. During the next step, two primary types of feature extraction are supported: relative plant mask areas or pod count estimation. Mask extraction is performed either with SAM3 using a text prompt or with the data-distilled SegFormer model. Pod counting is performed with trained P2PNet.

Using SAM3-generated masks as pseudo labels to train a more lightweight model is a natural step to reduce resource consumption for an edge-oriented solution. As presented earlier, SAM3 produced acceptable results on most scenes but sometimes displayed instabilities, which motivates some kind of filtering before distillation. Visual inspection of common SAM mistakes discovered that due to the high frame rate (90 FPS, downsampled to 9 FPS), adjacent images inevitably shared most of the plant area. SAM3 would then sometimes miss parts of the canopy or over-segment ground near the stem, which introduced noise to the relative area signal across video frames. This made basic rule-based filtering viable. The following rules were implemented:

1. Absolute area threshold: too low values indicate either incomplete view (e.g., there are almost no plants in the scene) or severe segmentation error.
2. Relative difference threshold: when some plant fails to segment across adjacent frames, it usually introduces a sudden drop in relative area. Similarly, when near-step ground is segmented by mistake, it also contributes to a difference in total mask area, which can be ruled out by thresholding such fluctuations.
3. Median Absolute Deviation: a more complex approach to handle temporal instability: assuming small position shifts across frames, when area in the current frame drifts significantly from a moving median, it might indicate that something went wrong and the current frame should probably be discarded.

Adjusting thresholds for these heuristics involves trade-offs between dataset size and quality. Using this method, a dataset with roughly 4000 training samples and 3000 validation samples was constructed. Each sample features a depth-filtered RGB image as input and a binary plant mask asthe target. Then, as a part of a separate study, SegFormer model is trained on this dataset, with Intersection over Union (IoU) plateauing around 0.85. Upon manual error inspection, it turned out that smaller model actually generalized beyond noisy targets introduced by insufficient dataset filtering on some samples, thus limiting achievable score value. It is further justified by different model sizes resulting in very similar IoU values, as shown in Table 3.

Once relative mask area or pod count is extracted from images, it forms a scalar time series across the source video. The next task is then to convert this signal to a scalar score that can be used for ranking. The following constraints and limitations had to be respected:

1. In our general setup with no reference frames and unknown video duration, there is no way to robustly sample non-overlapping plant scenes from it. The entire video should be processed regardless of its length, and the algorithm must be size-agnostic.
2. Consequently, the number of plants present in the bed is unknown, and severe distortions and shape ambiguity render plant detection methods unviable, so there is no way to robustly extract per-plant features such as pod or bean count, spatial dimensions, etc.
3. Stitching across frames to produce a continuous panorama-like scene is technically infeasible due to fisheye distortions. Camera calibration discards 70% of the scene.

The task was therefore simplified to the aggregation of scalar signals across frames in some length-invariant way. One simple method would take the mean or median of a scalar distribution across frames. Alternatives such as per-plant detection and classification or plot bounding-box detection require a method to robustly sample key frames with minimal spatial overlap. This is a complex problem on its own and thus was not further developed in this work.

## 4. Results

This section contains descriptions of the proposed methods for ranking soybean plots. Two primary pipelines are considered: plant segmentation and pod detection and counting, both sharing a background subtraction step via depth estimation. Ranking experiment design is discussed. Pod detection and mean aggregation are shown to achieve the strongest ranking performance among the considered methods based on the evaluation metrics.

In order to prove that the proposed ranking algorithm is actually working, a dataset with natural ordering should be processed with the proposed pipeline to compute ranking metrics. To build this dataset, first, a collection of soybean plants was manually segregated into three groups (pseudo-classes): average (or medium), small (or below expectations) and large (or exceeding expectations) based on the number of pods and overall plant size. These classes were then used to create scenes of varying plant density to imitate different plot qualities. Plants are put into plots, then a camera mounted to a trolley records video footage. Collected videos are processed, and final area distributions and mean statistics are obtained, as shown in Figure 13. It should be noted that camera position needs to be fixed to faithfully rank multiple scenes, as area distributions can easily drift as camera height changes. Experiments show that the optimal camera height ranges from 35 cm to 43 cm from lower metal frame; values exceeding this range might not capture the plant entirely. Area distributions for scenes constructed with plants from one of the three classes are displayed in Figure 14.

Three scenes generated entirely from single-class plant samples prove that the area distribution is a meaningful quality signal. However, a larger-scale experiment is required to collect more evidence. Another natural and easily implemented way to build a dataset for this experiment is shown in Figure 15. Six soybean plots are used as placeholders for plants in one of the three pseudo-classes from the previous experiment (or no plants in the empty case). Starting from a set of plots with some classes, on each iteration, plants in one plot are replaced by plants from a strictly higher-quality pseudo-class. For example, an empty plot can be replaced with anything, small plants can be replaced with medium or large ones, and medium plants can be replaced by the large pseudo-class only. This yields a strictly monotonic sequence that can be used to compute ranking metrics. In the experiments, metrics such as mean area and mean soybean pod count are extracted and directly used to rank scenes.

To further aid reproducibility, an additional scene is generated for each plot sequence by swapping one or two pairs of plots. This allows measurement of both how well the models separate ranks and how stable they are to perturbations within the same rank. A total of 11 ranks with 2 samples per rank are collected, producing 22 samples in total. This is repeated for indoor and outdoor scenes to inspect the performance gap across these domains.

Violin plots with area and detected soybean pod count distributions across indoor and outdoor scenes are shown in Figure 16 and Figure 17. As observed earlier for the three-pseudo-class case in Figure 14, rank-dependent probability mass shifts are still present as more ranks are introduced. Figure 18 and Figure 19 show how different scalar metrics rank videos from indoor and outdoor datasets, respectively. All figures feature apparent negative trends but vary in intra-rank gaps and monotonicity. For good ranking, minimal gaps between scenes of the same rank and strong monotonicity between ranks are required. Mean pod-count achieves the strongest ranking performance among the evaluated features for both indoor and outdoor scenes. Mean plant area is also a strong quality proxy. In contrast to the initial hypothesis, distribution medians proved to fit this task worse than means. Table 4 summarizes the ranking quality for all tested methods on indoor and outdoor scenes, respectively.

## 5. Discussion

This work addresses automated soybean plot ranking from close-range RGB fisheye videos—a task where conventional object detection and instance segmentation fail due to severe stem overlap, mature pod clustering, camera barrel distortion, and high annotation costs. Our primary contribution is a video-level pipeline that bypasses per-plant instance parsing entirely, relying instead on aggregate frame-level signals: relative plant area (via depth-filtered segmentation) and total pod counts (via point-based keypoint detection). By integrating monocular depth estimation (Depth Anything 3), zero-shot segmentation (SAM 3 distilled into SegFormer), and supervised point counting (P2PNet), our workflow achieves robust quality ranking without requiring costly bounding-box annotations. The practical significance of our approach is twofold. First, we provide systematic empirical evidence that general-purpose vision models (YOLO, RT-DETR, Grounding DINO) perform poorly under high fisheye lens distortion and cluttered background settings, challenging the assumption that standard detectors transfer directly to agricultural phenotyping. Second, we demonstrate that aggregate pod count—a metric validated by agronomic literature as a critical yield proxy—reliably separates soybean plots of differing quality, achieving strong Normalized Discounted Cumulative Gain (NDCG) and rank correlation metrics. On our controlled ranking dataset, mean pod count and area-based features are used to reliably separate plots of different quality, achieving high NDCG and correlation statistics. Our method therefore offers a practical tool for breeders to accelerate selection cycles while reducing manual labor.

Several constraints qualify these findings. First, the evaluation dataset was acquired in a controlled indoor setup with manually grouped planters rather than measured field yield. Second, the pipeline relies on fixed camera-to-trolley geometry and does not yet perform pod-to-stem tracking, preventing direct per-plant yield attribution.

Subsequent work will extend the discussed method by developing stem tracking algorithms for pod-to-plant association and leveraging multi-view point clouds via Depth Anything 3 for better plant separation. We will also validate ranking metrics across outdoor plots featuring diverse genotypes and test the vision stack with autonomous field rovers to eliminate camera height sensitivity.

## Figures and Tables

**Figure 1:**
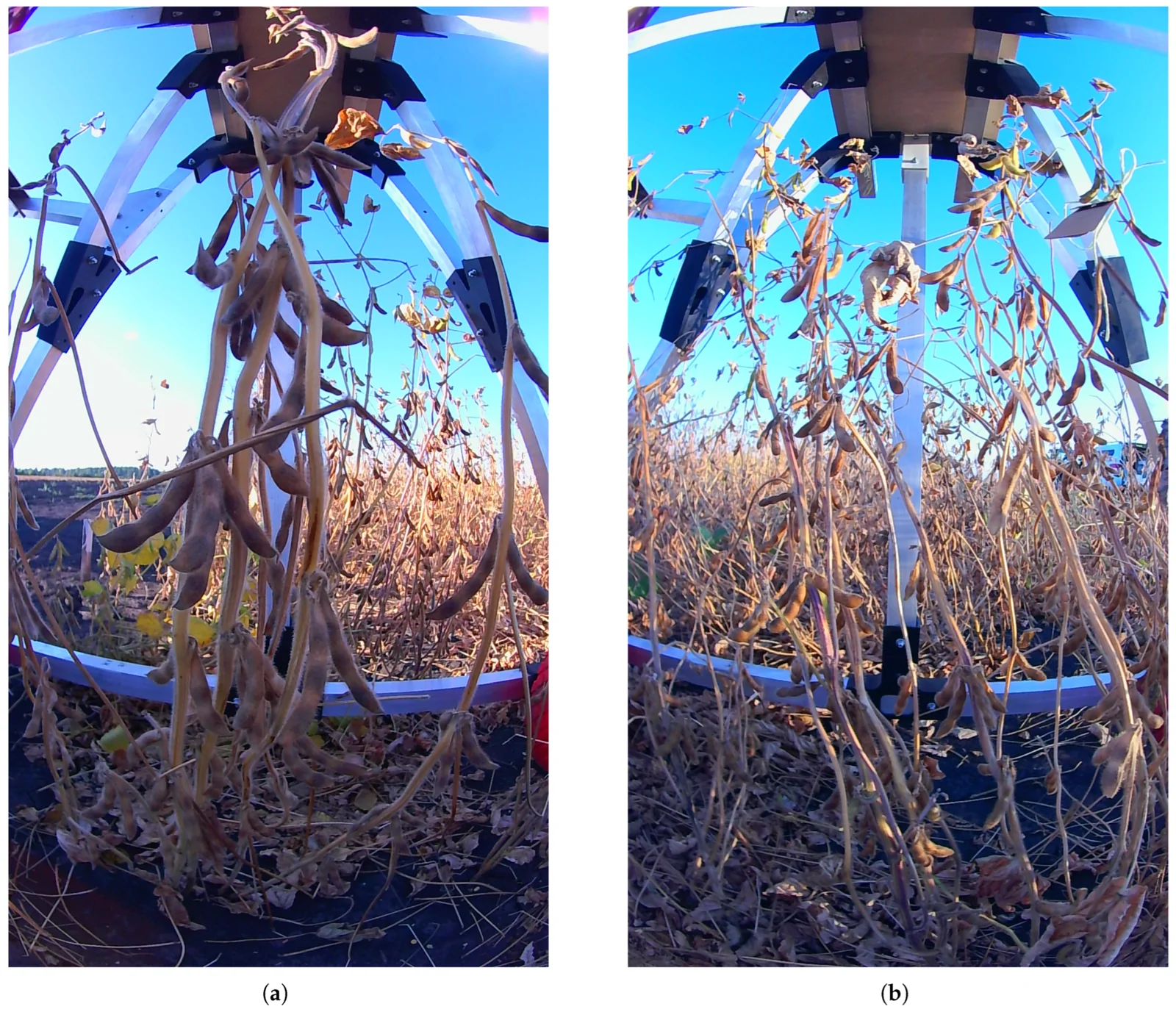
Soybean plot imagery from open field that displays key challenges addressed by this work. (**a**) Severe fisheye distortions near frame borders. (**b**) Visual clutter and ambiguous plant boundaries.

**Figure 2:**
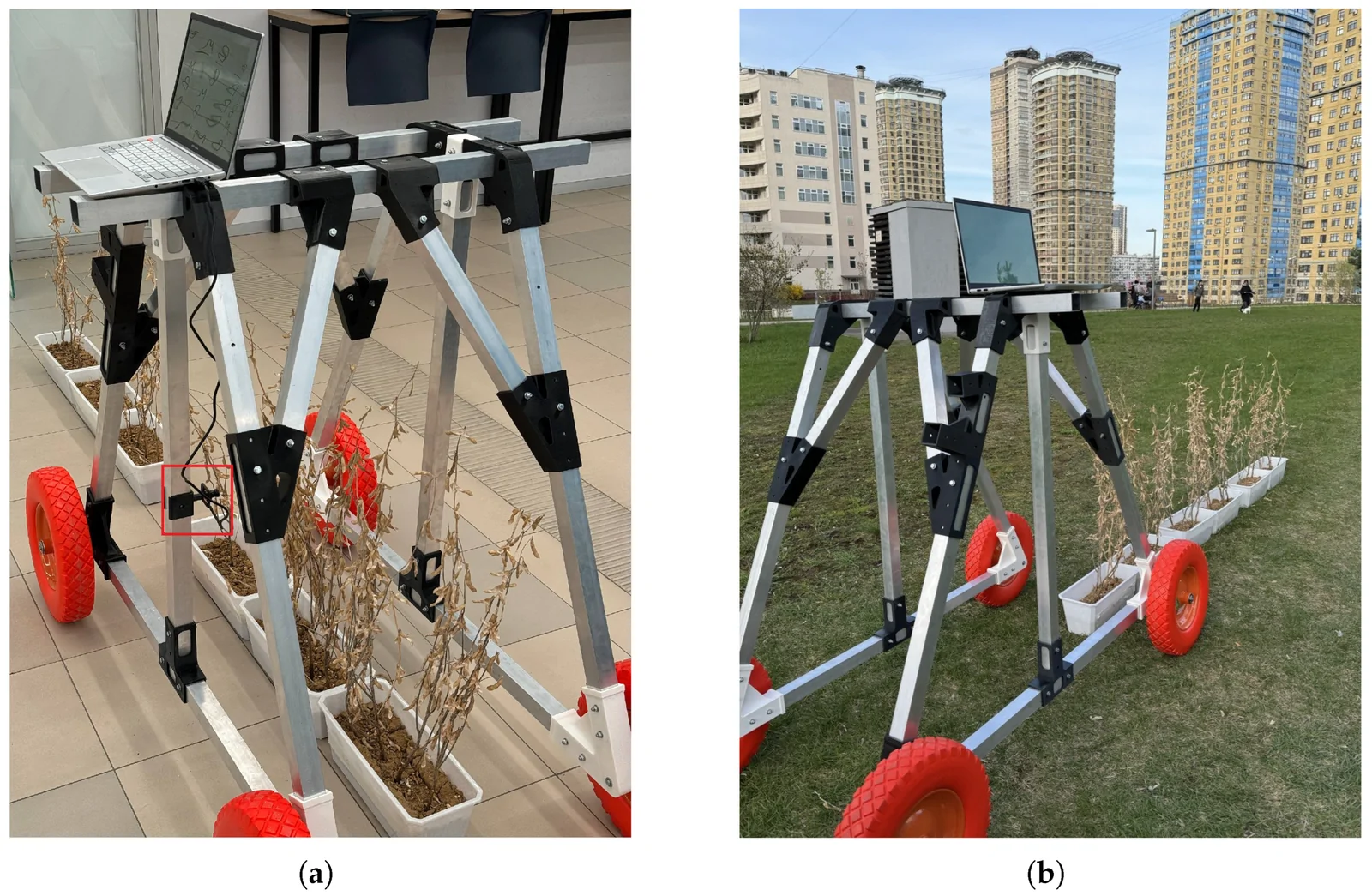
Mobile trolley developed for soybean phenotyping project, featuring high clearance and 160° wide attachable USB camera for data collection (designated by a red box). (**a**) Indoor view. (**b**) Outdoor view. Best viewed in color.

**Figure 3:**
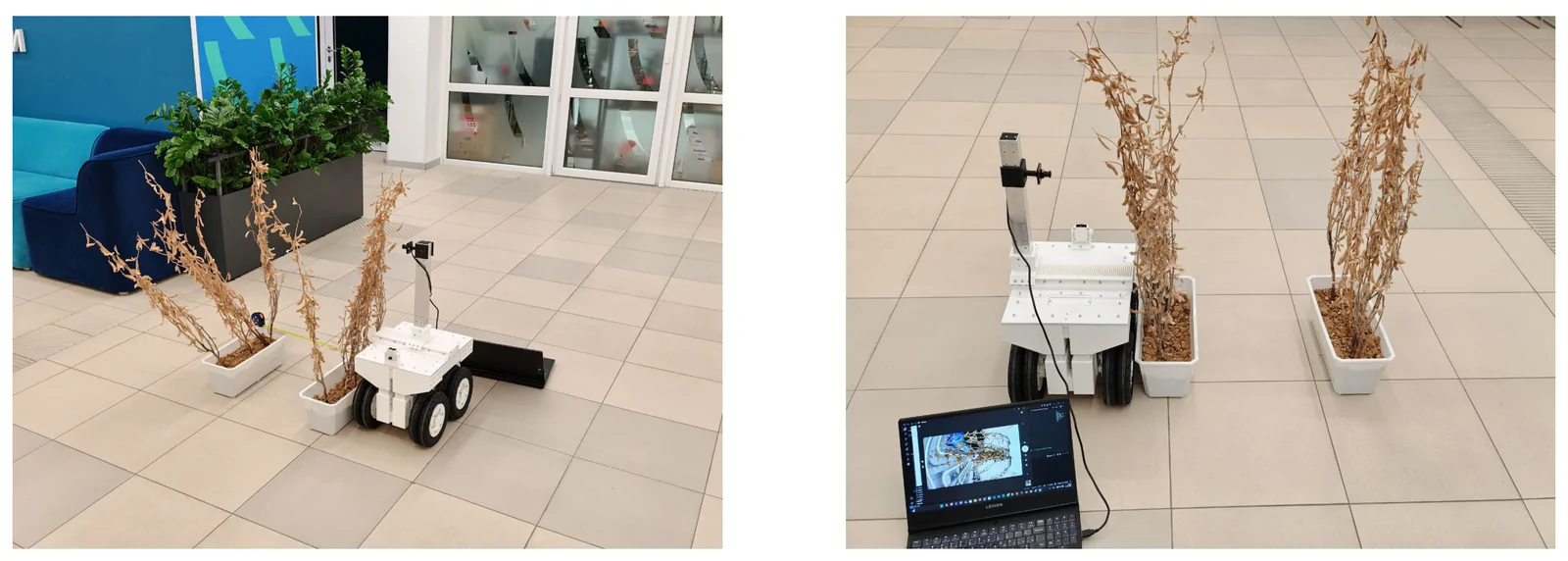
A small rover bot built in a separate study applied to soybean plant data collection with the same USB camera. Autonomous drone usage is a promising direction for further development.

**Figure 4:**
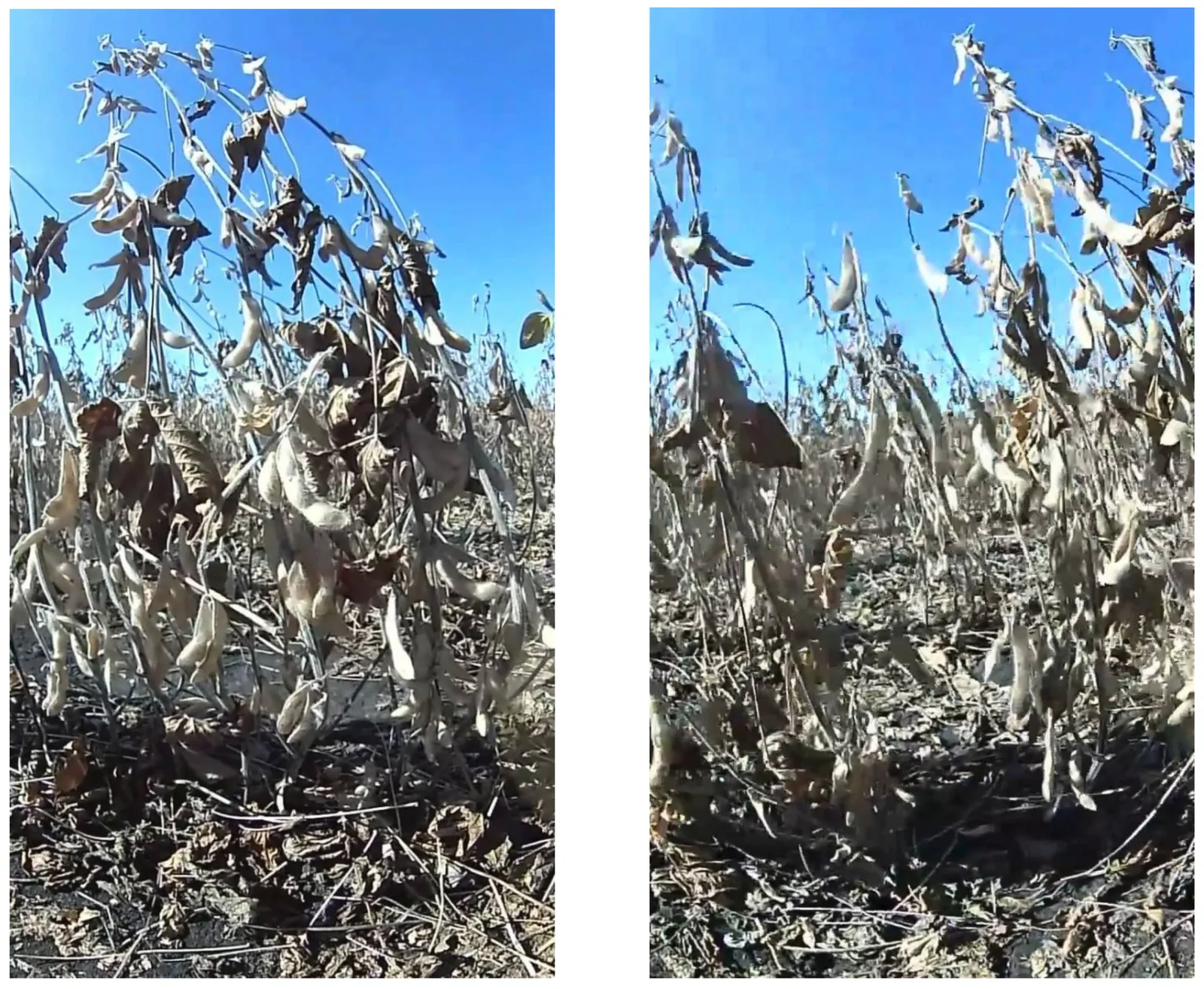
Examples of soybean plant images obtained by 160° wide camera in outdoor field conditions. Color distortions are introduced by a missing infrared filter. Best viewed in color.

**Figure 5:**
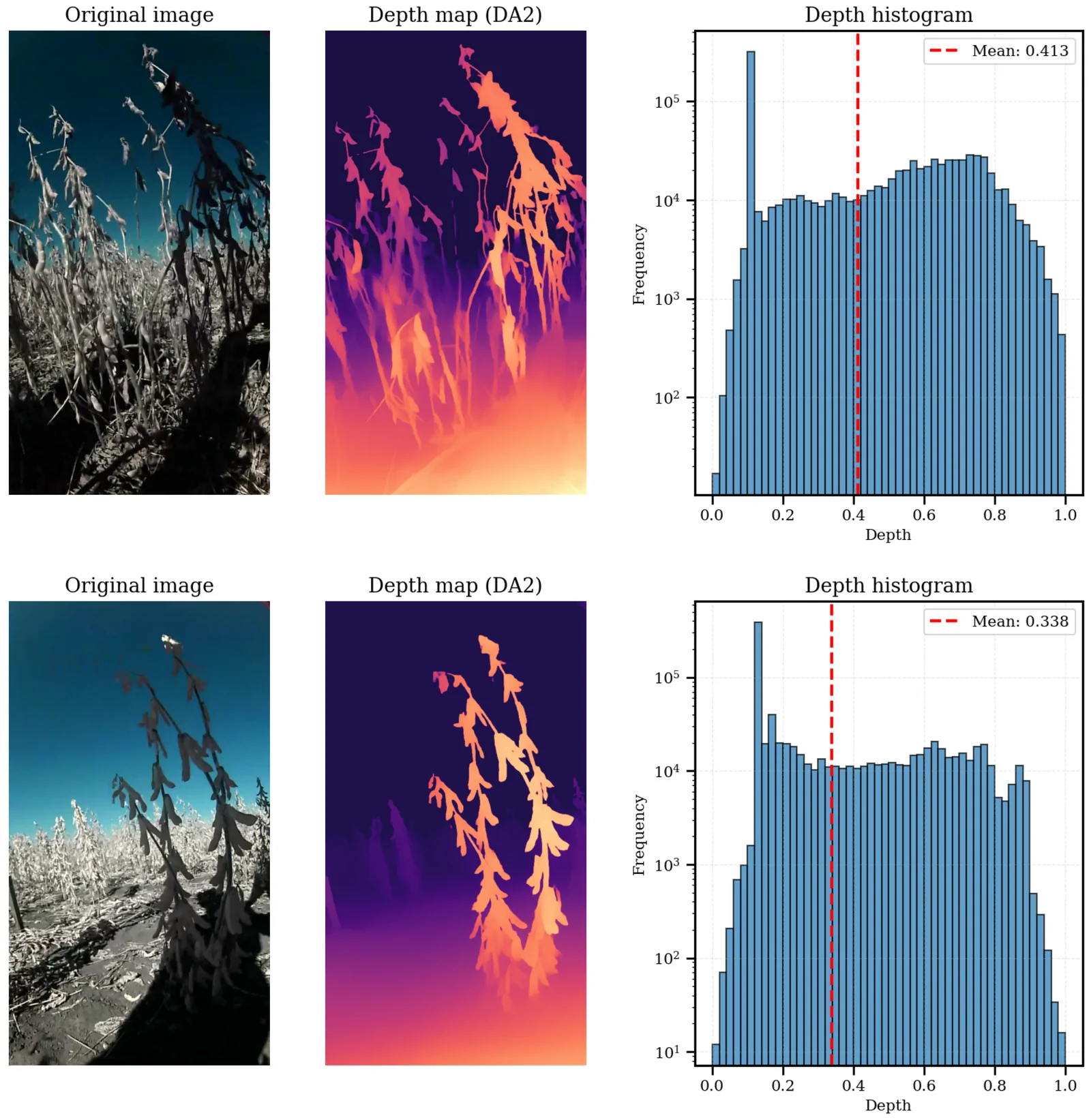
Examples of depth estimation and depth distribution histograms obtained with Depth Anything 2 [25]. Best viewed in color.

**Figure 6:**
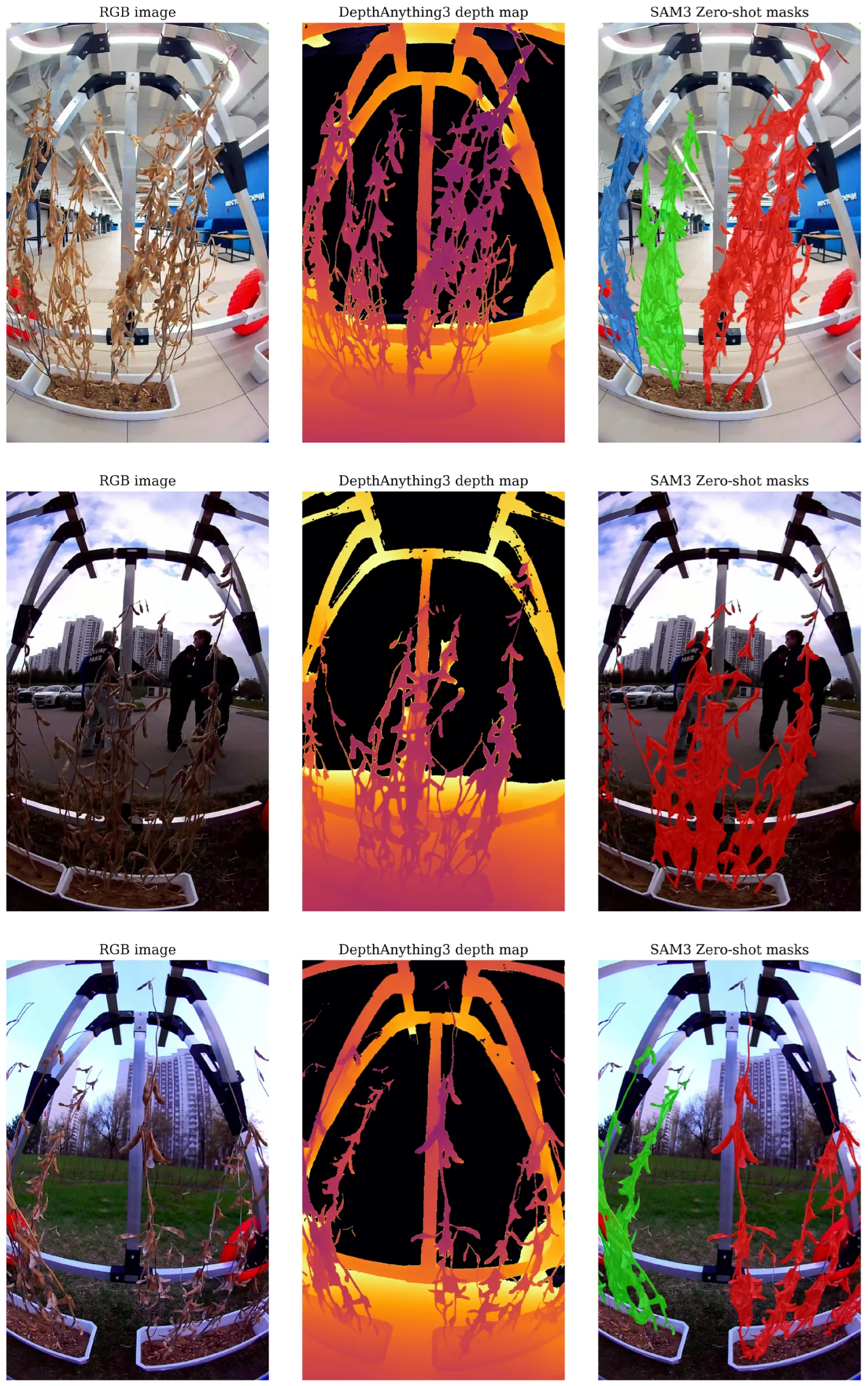
Examples of depth estimation and zero-shot segmentation of plants in various conditions (indoor, outdoor). Colors in the right column represent distinct instance masks. SAM3 fails to provide consistent plant-level predictions yet, thus we merge them into a single mask per frame. Best viewed in color.

**Figure 7:**
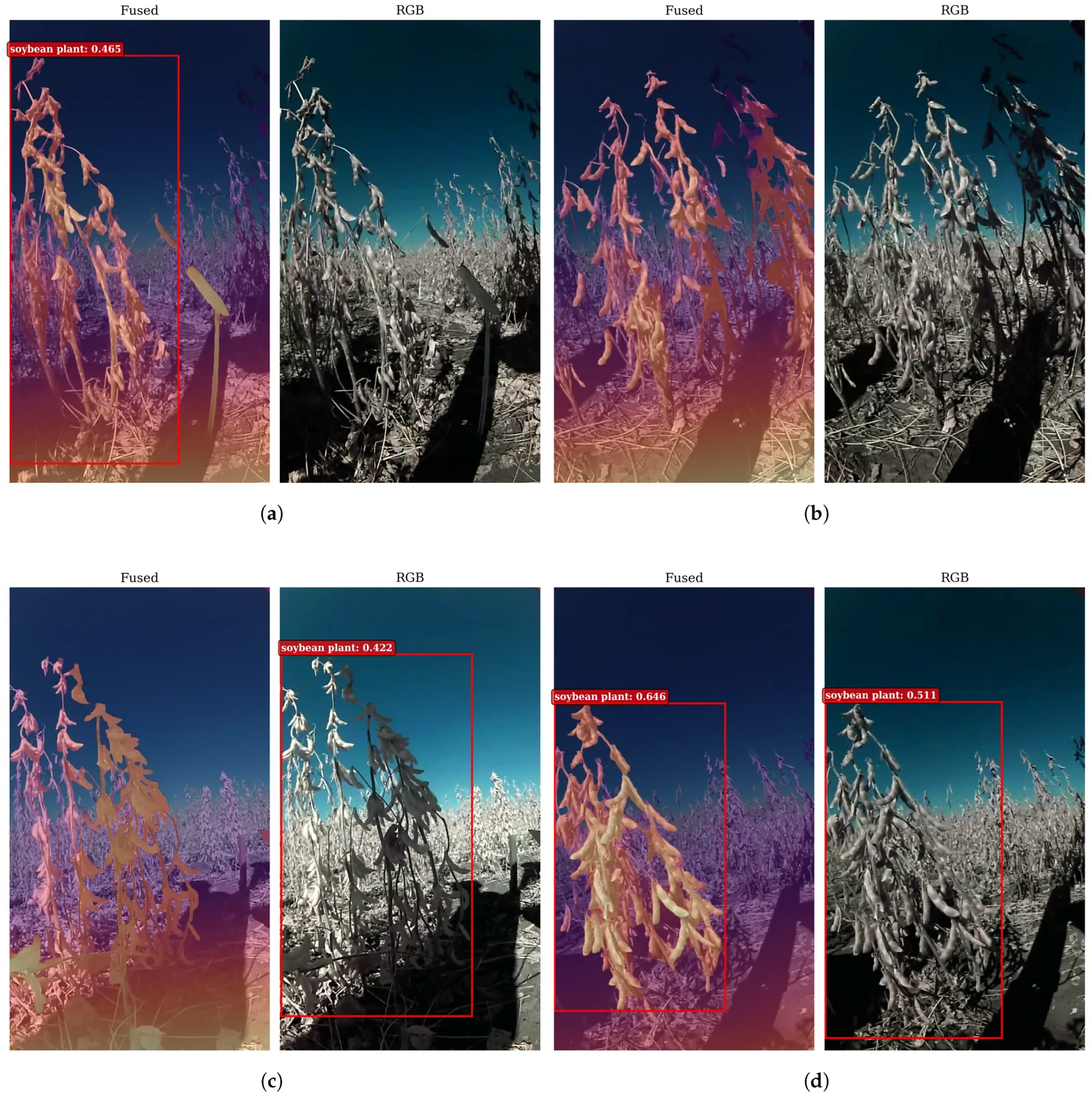
Examples of zero-shot detections produced by Grounding DINO detector [14] with a confidence threshold of 0.4. Detections are unstable across scene types, even when alpha-fused with the depth map. (**a**) Plant only detected in RGB-highlighted frame. (**b**) No successful detections. (**c**) Plant only detected in original RGB frame. (**d**) Detected for both images, depth-highlighted has higher confidence. Best viewed in color.

**Figure 8:**
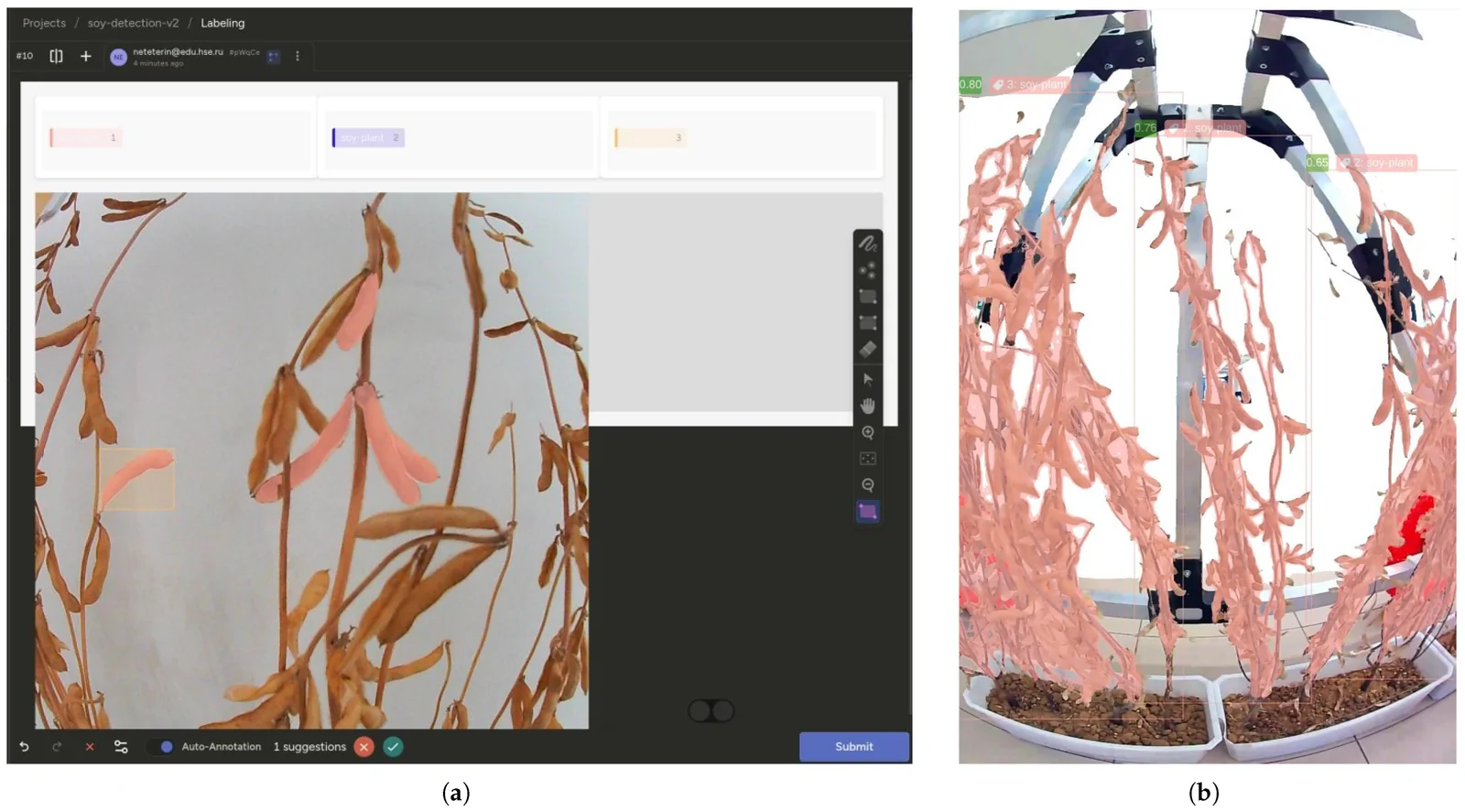
Interactive annotation process in LabelStudio [26] with Segment Anything Model 2. (**a**) Manual box prompts (yellow) produce precise pod masks (pink). (**b**) Plant masks generated with SAM2 and corresponding box prompts. Best viewed in color.

**Figure 9:**
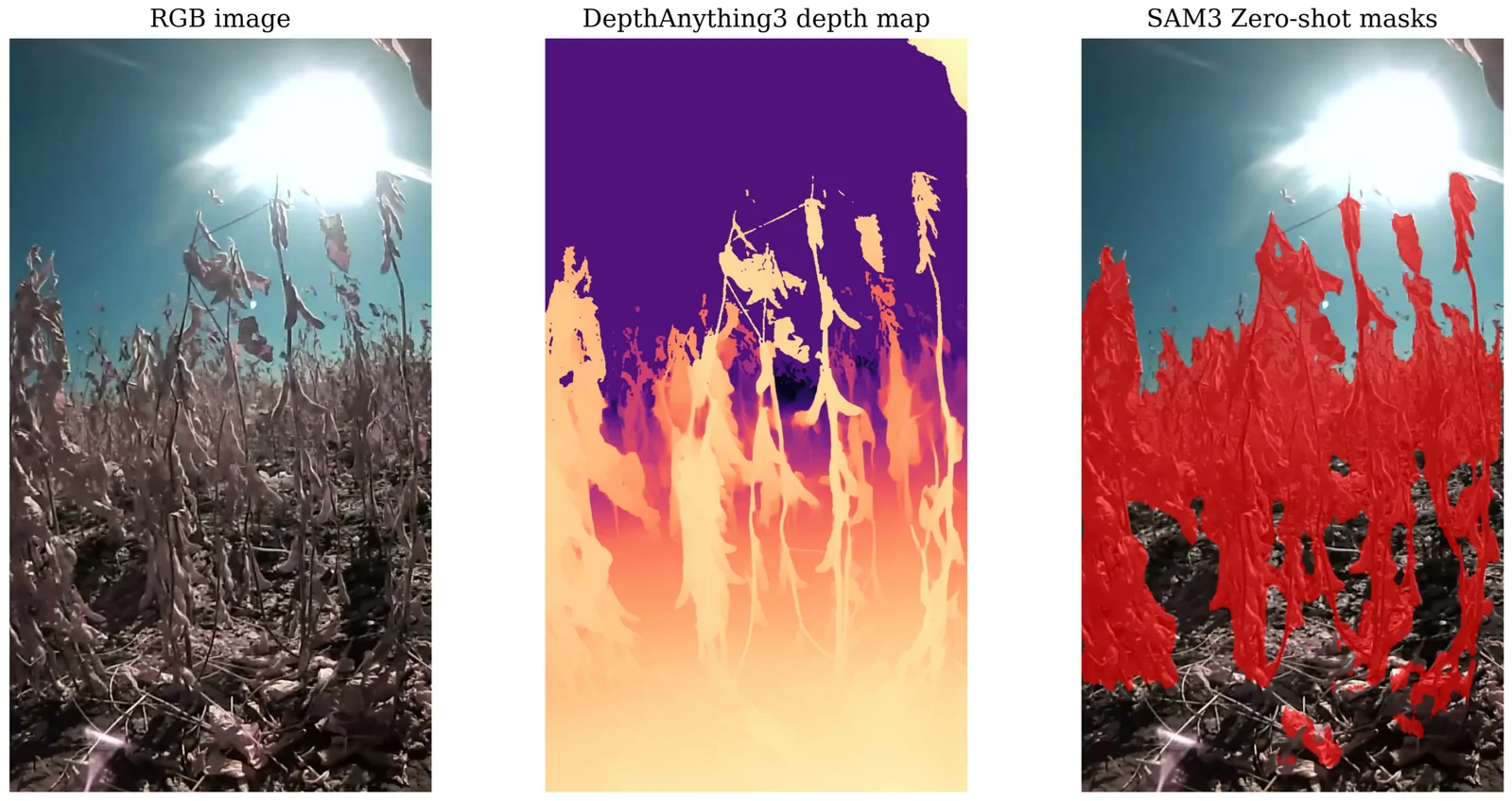
SAM3 failing to segment plants. (**Left**): sample image collected from soybean field for this study, featuring several plants in the foreground and highly occluded background; (**center**): depth map generated by Depth Anything 3 model [23]; (**right**): zero-shot mask generated by SAM3 [12] using a text prompt “individual soy plants in the foreground”. Despite there being at least 4 clearly separable plants, SAM3 fails to distinguish between them and generates a single oversized mask.

**Figure 10:**
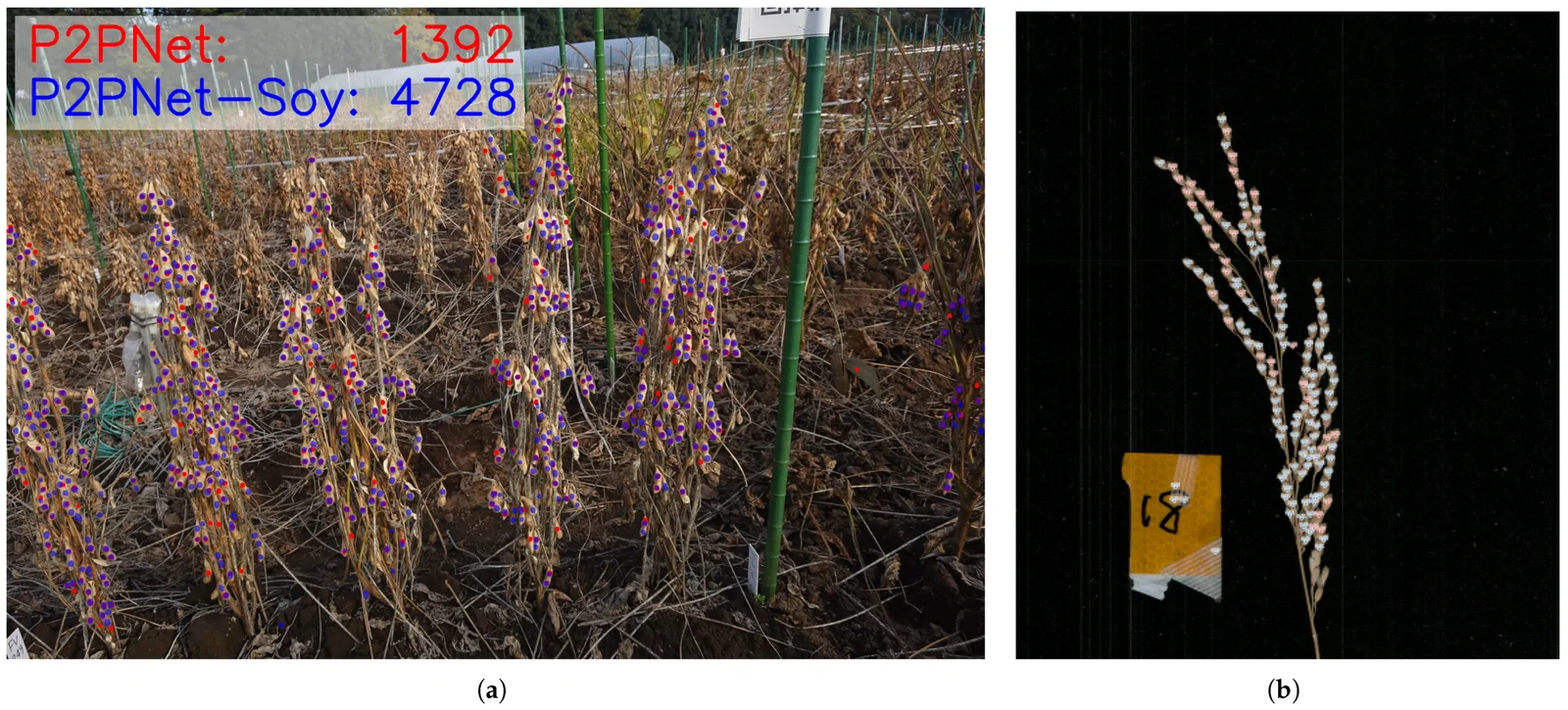
EasyP2PNet applied to agricultural problems. (**a**) Soybean counting on a high-quality in-field image. Red dots are seed point predictions of the original P2PNet, and blue dots are seed predictions of an improved model from [21]. (**b**) Dual-class grain counting in rice panicles in [22]. Red and blue dots mark filled and unfilled rice grains respectively. Both scenes are substantially different from our setup with less overlap and no distortions. Best viewed in color.

**Figure 11:**
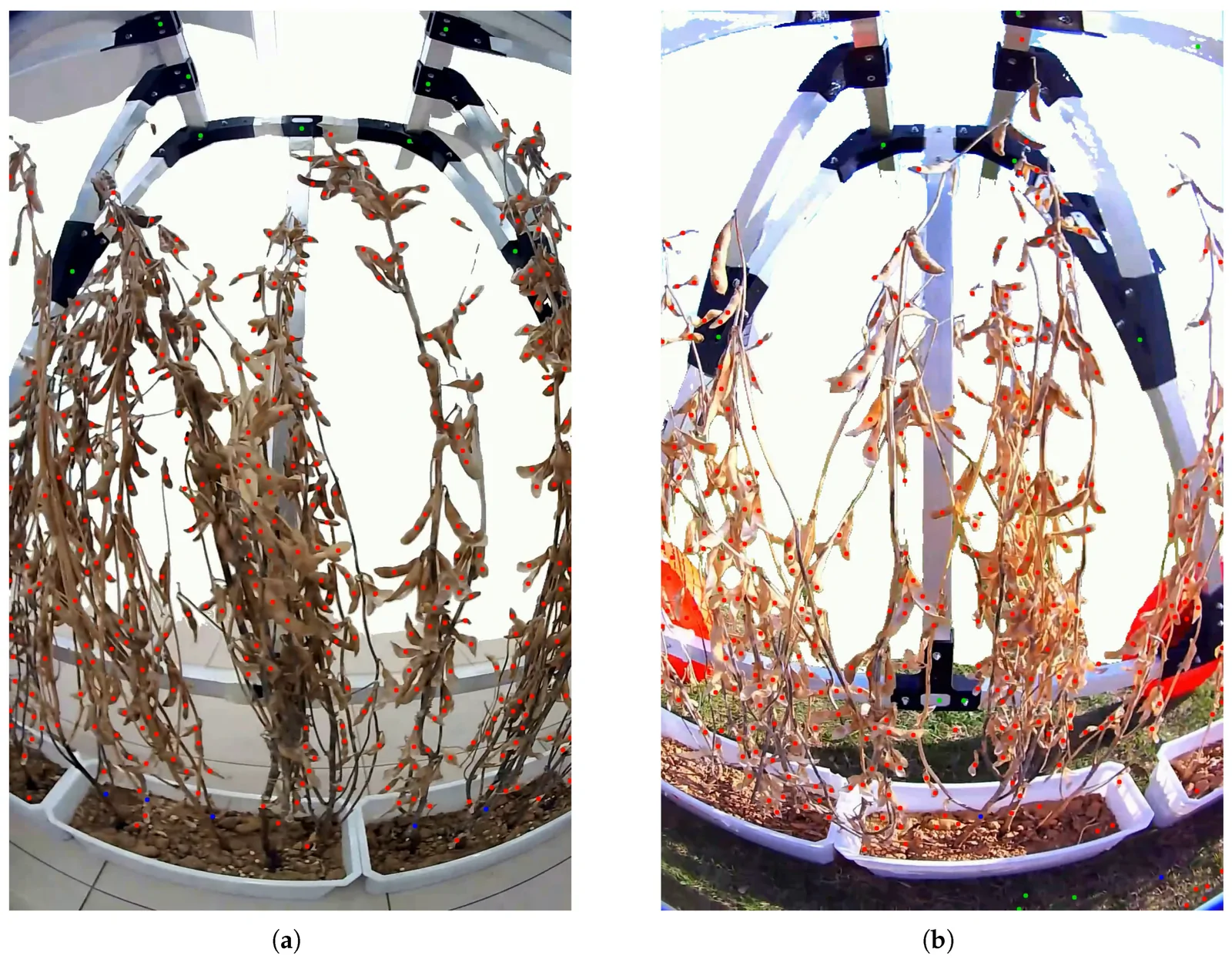
P2PNet inference after background removal: (**a**) indoor scene, (**b**) outdoor scene. Red, blue and green dots mark pod, plant stem and trolley frame keypoint predictions, respectively. When applied to an outdoor scene, the model fails to detect some stems and produces false positives for all classes in background grass regions, presumably due to the training set only featuring indoor scenes with consistent illumination and no shadows. Best viewed in color.

**Figure 12:**
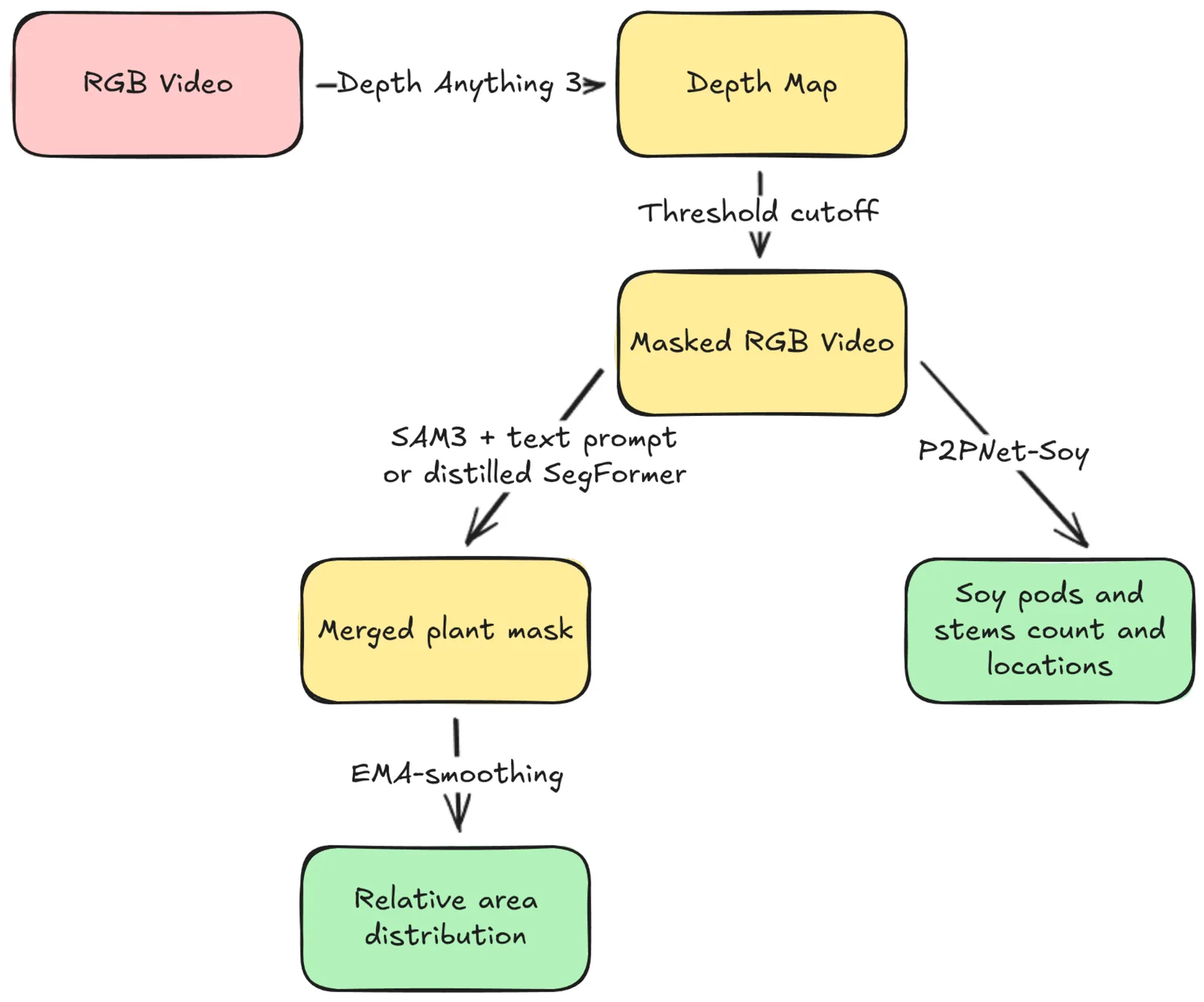
Proposed soybean plot scoring pipeline. Pipeline inputs and outputs are colored in red and green, respectively. Intermediate computations are colored in yellow.

**Figure 13:**
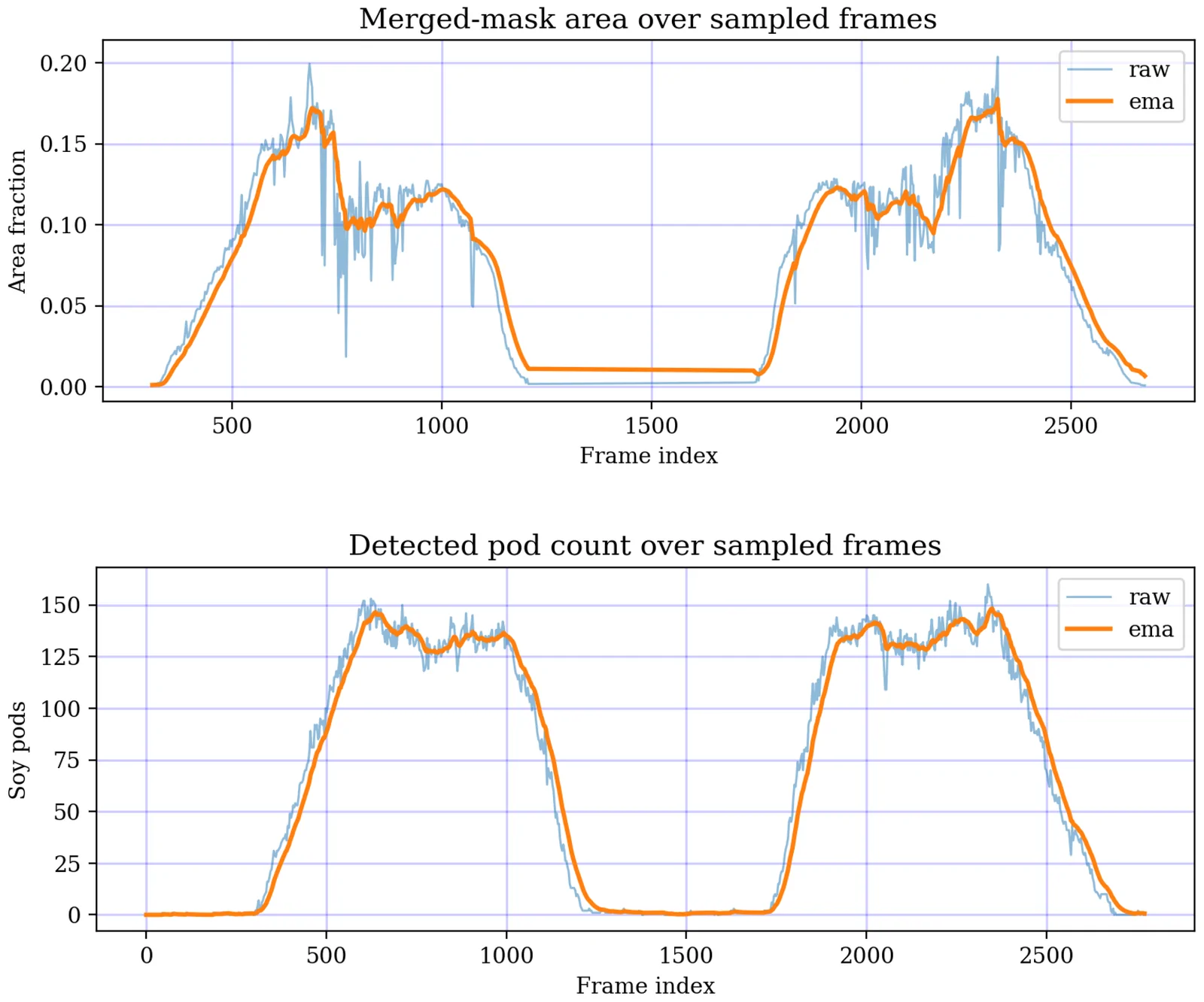
Merged mask area and detected pod count series extracted from the same video. Traces are nearly horizontally symmetrical around frame at index 1500 due to camera recording the scene in both directions. Oscillations in raw area series highlight segmentation instability, mitigated by EMA smoothing.

**Figure 14:**
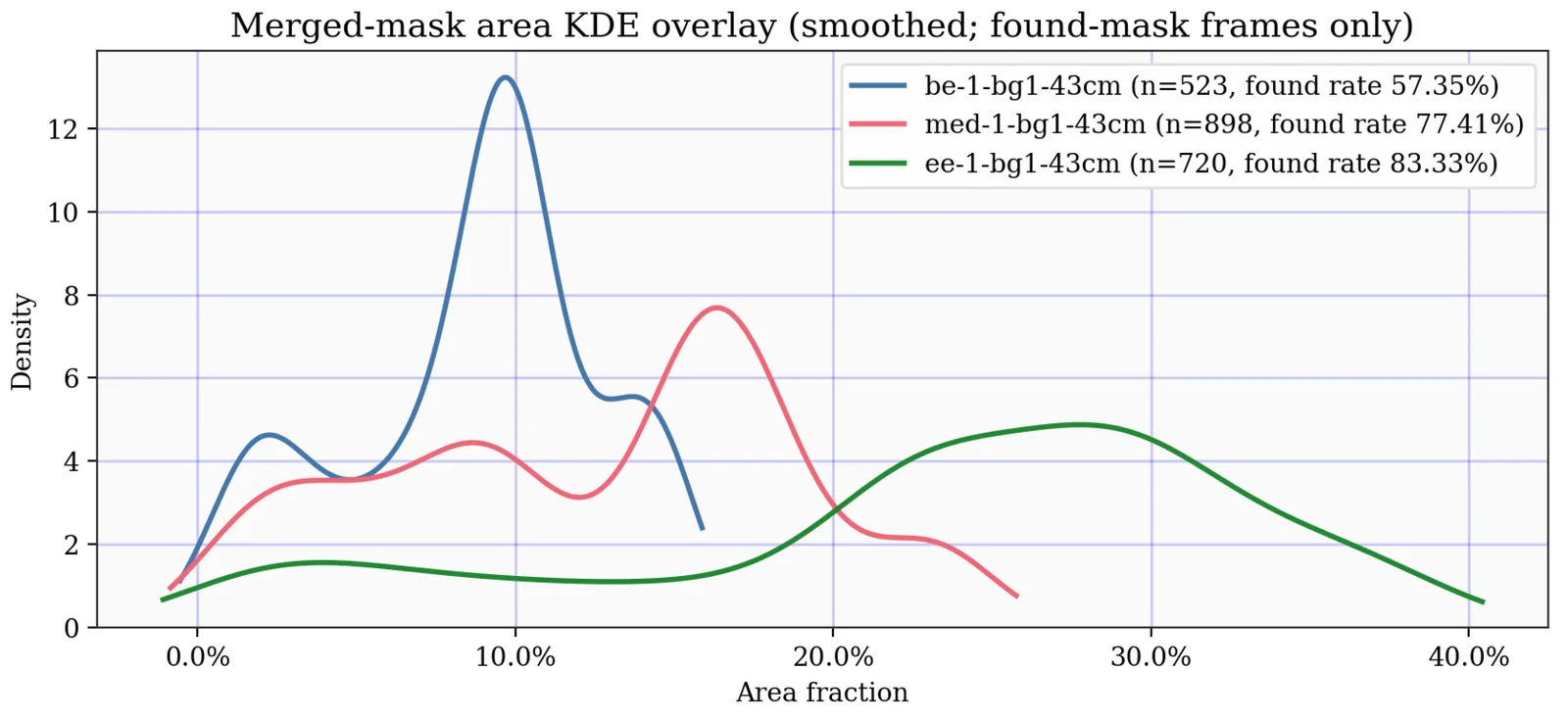
Area distributions collected for scenes constructed with plants from a single pseudo-class (below expectations, medium and exceeding expectations), taken with the same camera viewpoint and background. Distributions show class-dependent shifts in probability mass.

**Figure 15:**
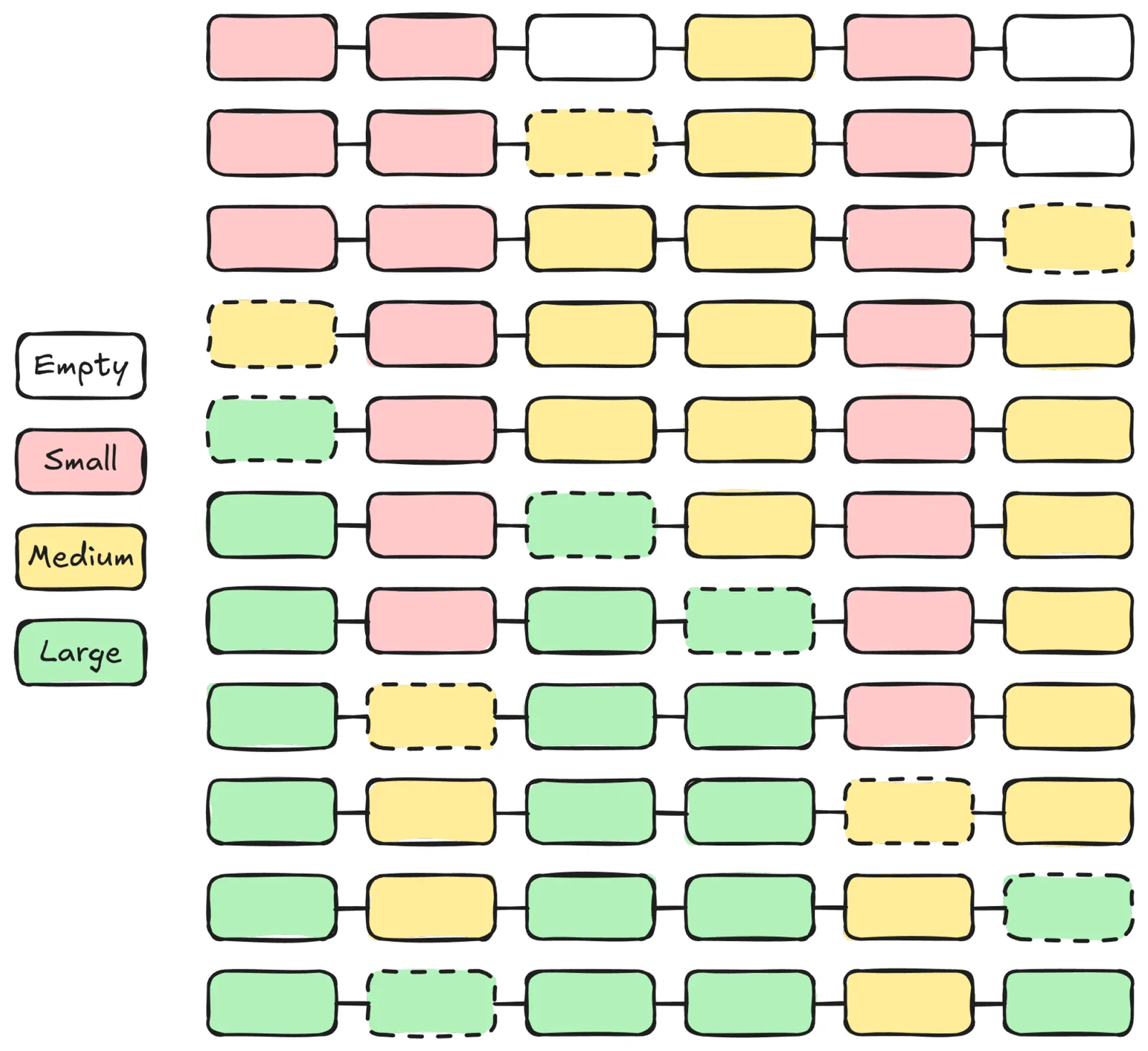
Proposed algorithm to build ranking dataset with natural ordering using three pseudo-classes of plants and six plots. Starting from the top row, on each iteration, one plot (marked by dash lines) is replaced with a plot with strictly higher-quality plants, which ensures monotonicity. Best viewed in color.

**Figure 16:**
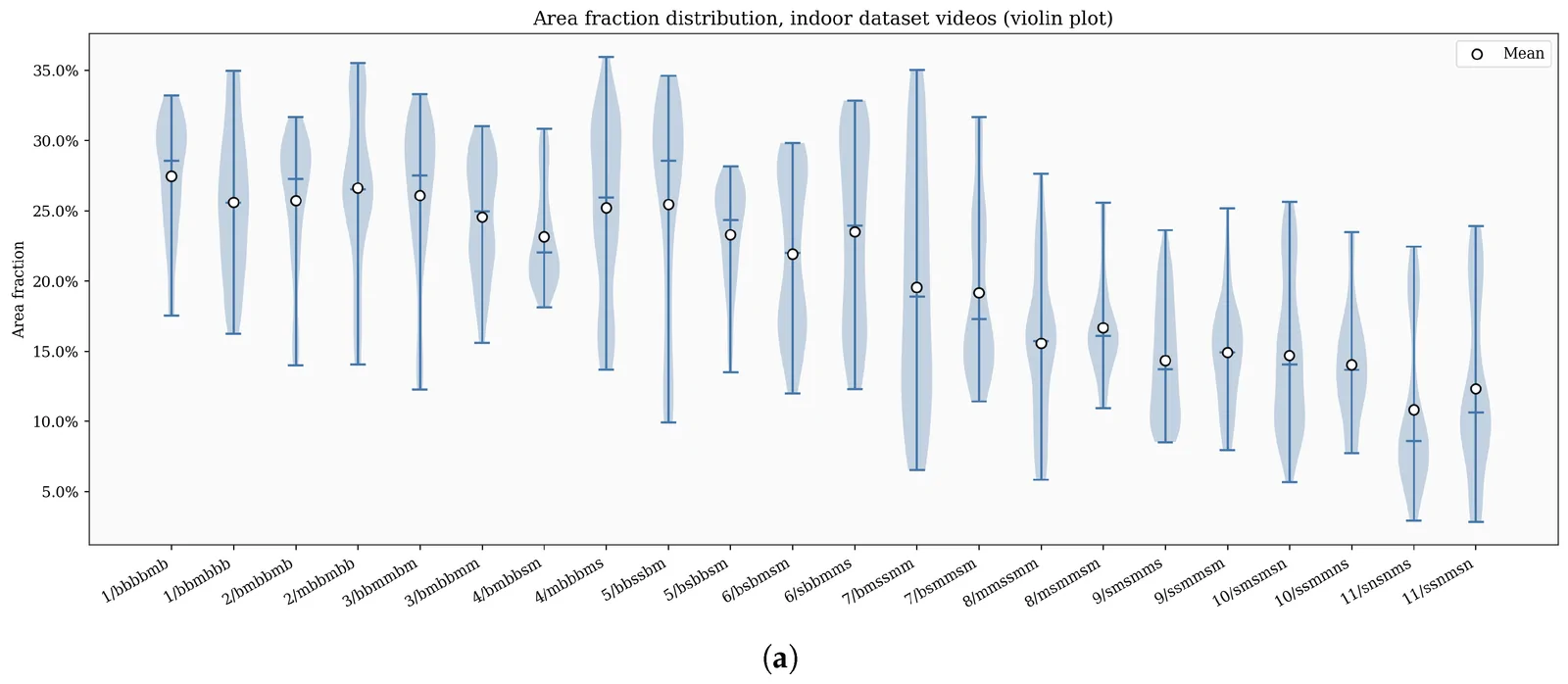

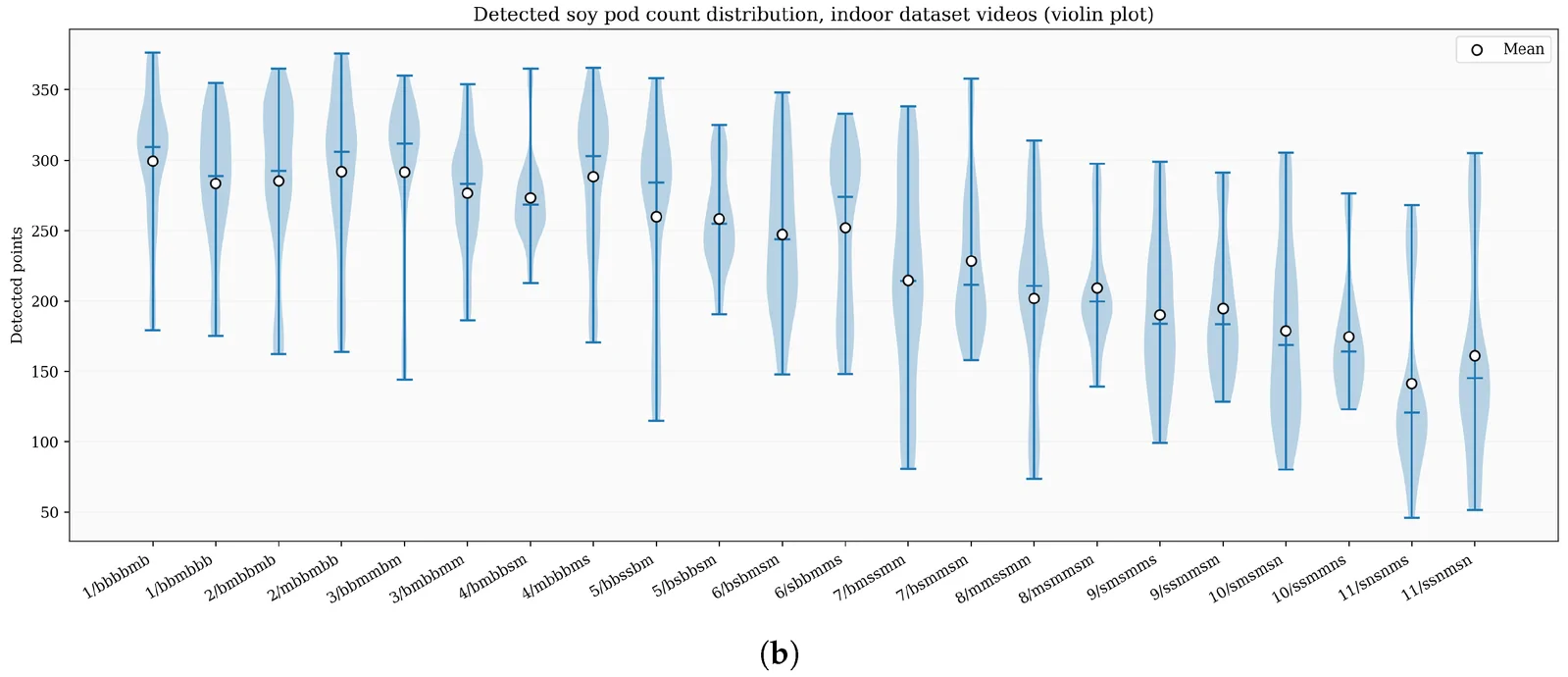
Violin plots for scenes from the indoor dataset: (**a**) relative plant mask area, (**b**) soybean pod count. Labels on the X-axis represent ground truth rank and class sequence in the sample, e.g., 1/bbbbmb is rank 1 featuring a sequence of 4 big class samples, a single medium sample, and another big class sample. General negative trend of distribution medians implies that rank-dependent shifts in probability mass persist, so these features remain usable as ranking signals.

**Figure 17:**
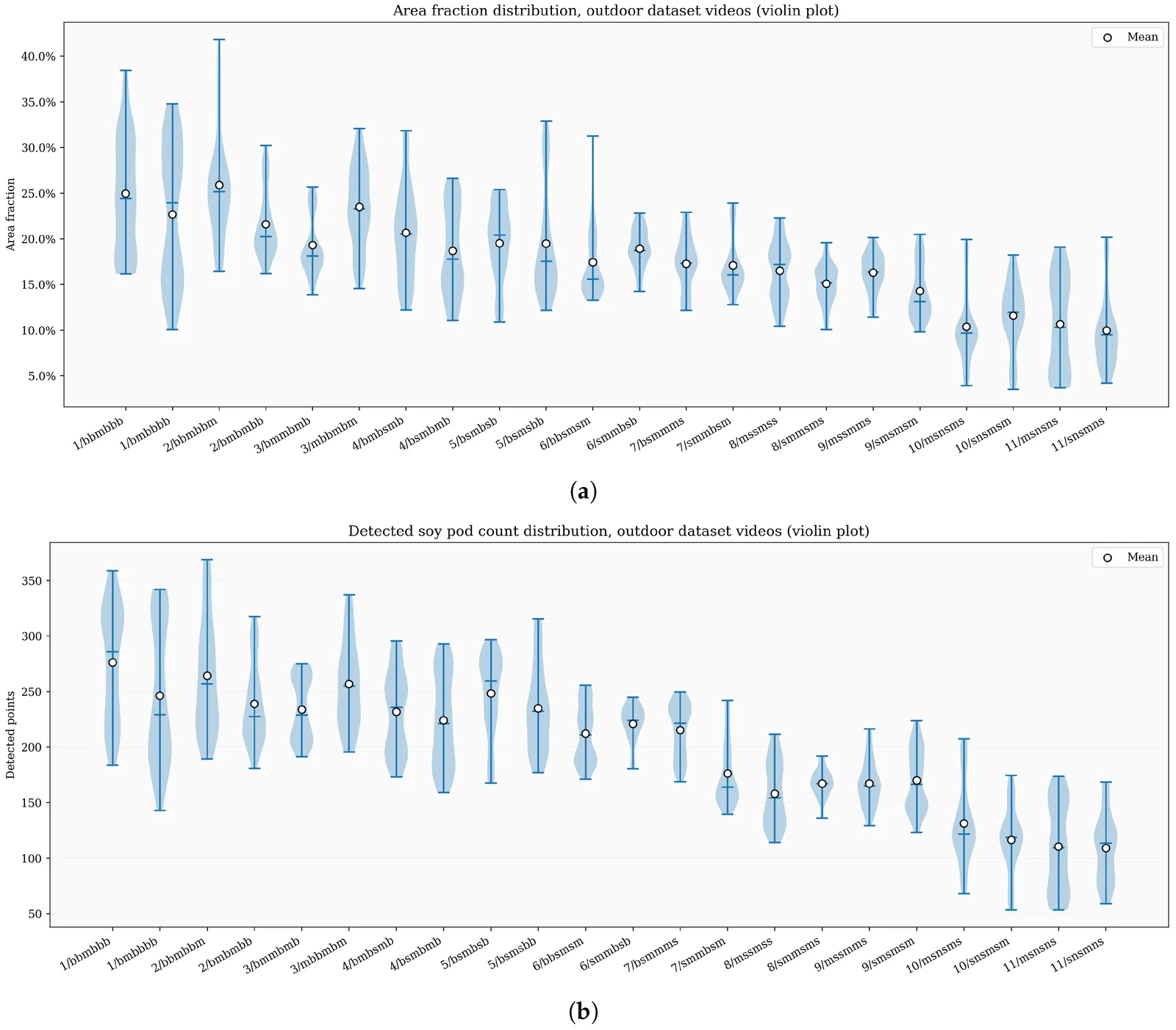
Violin plots for scenes from outdoor dataset: (**a**) relative plant mask area, (**b**) soybean pod count. Axis label notation follows Figure 16. The same rank-dependent negative trend is observed outdoors.

**Figure 18:**
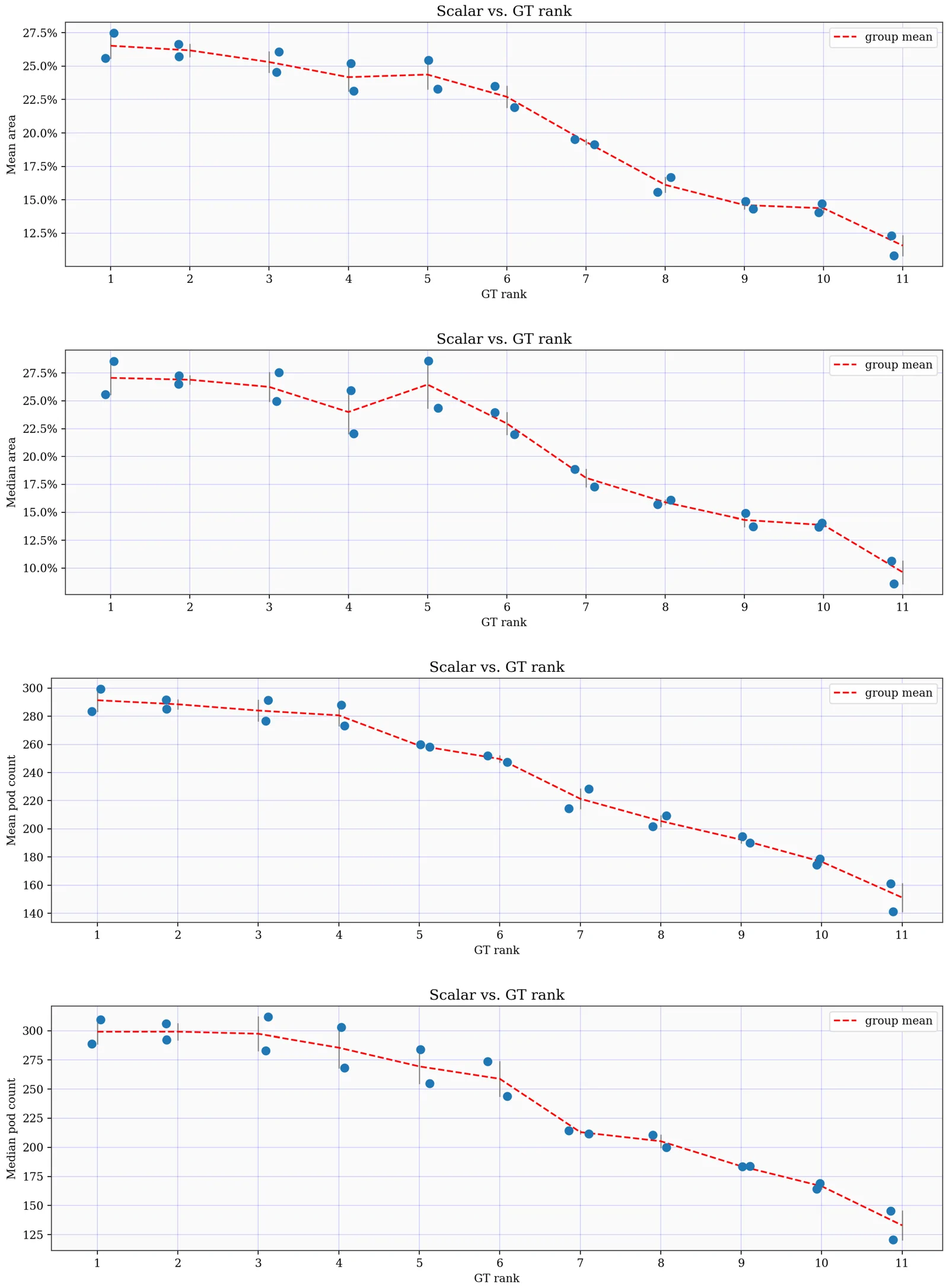
Indoor dataset ranking using scalar metrics and Ground Truth (GT). Blue dots represent two samples per rank, smaller intra-rank gaps are better.

**Figure 19:**
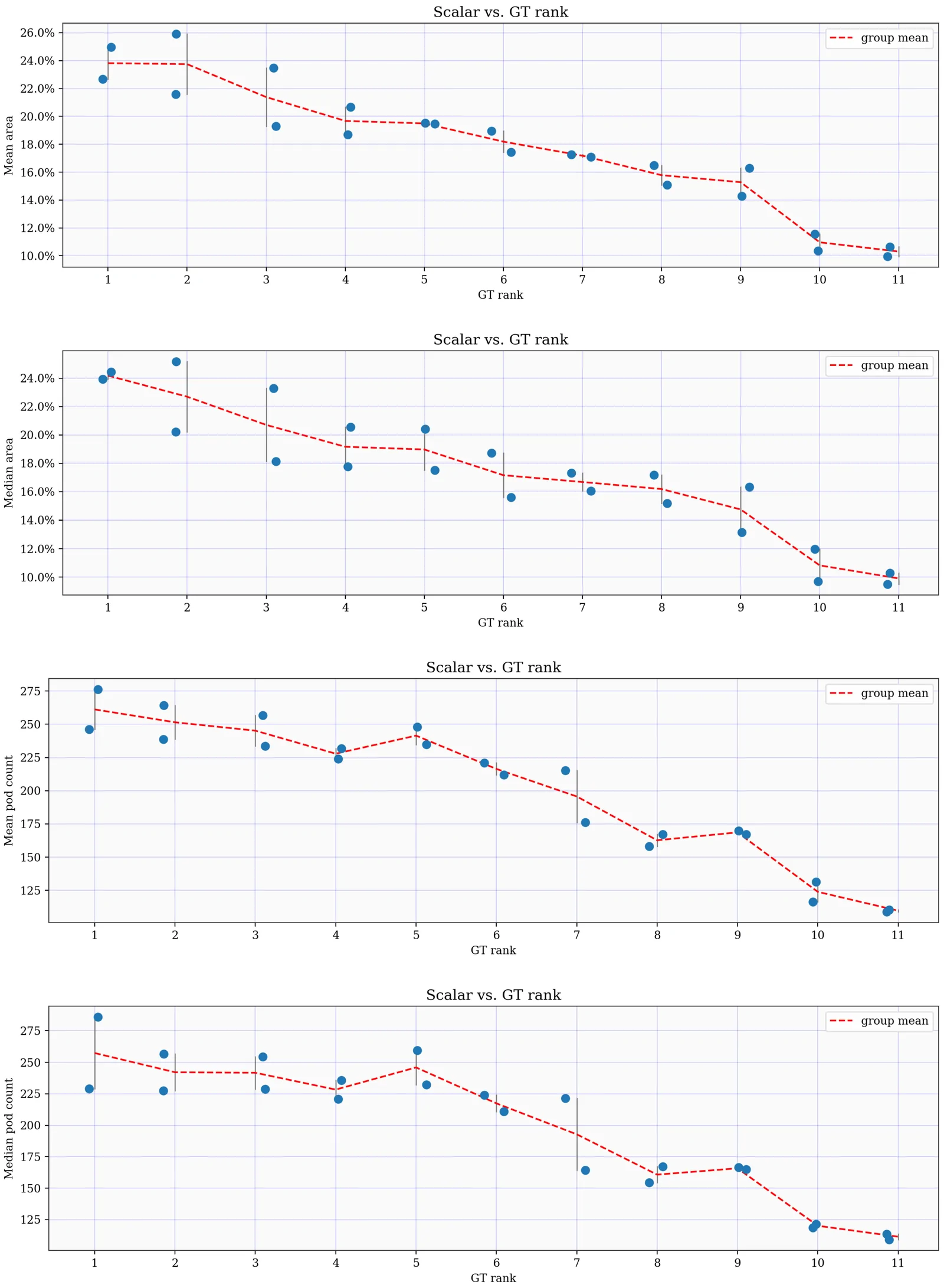
Outdoor dataset ranking using scalar metrics and GT. Blue dots represent two samples per rank, smaller intra-rank gaps are better.

**Table 1:** Evaluation metrics for models trained on soybean plant detection task. Low mAP@50-95 values highlight training instability and overfitting. Background removal slightly improves performance. Higher is better. Bold values denote best values achieved for each metric.

Model	Precision	Recall	mAP@50	mAP@50-95	Comment
YOLOv11m	0.687	0.639	0.694	0.306	RGB images
RT-DETR-L	0.735	0.647	0.688	0.330	RGB images
RT-DETR-L	**0.773**	**0.733**	**0.739**	0.357	Aggressive augmentations
RT-DETR-L	0.741	0.701	0.737	**0.370**	No background
YOLOv11m	0.760	0.668	0.734	0.323	No background

**Table 2:** P2PNet validation metrics for soybean pod counting. Bold values denote best values achieved for each metric.

Patch Size	MAE	MSE	F1 Score	Matched Point Distance MAE
256 (sampled from 384)	**2.78**	**3.64**	0.60	14.97
256	2.89	3.73	**0.61**	**13.79**

**Table 3:** IoU and model size comparison for SegFormer models fine-tuned on plant segmentation task from SAM3 generated labels.

Model	Parameters	IoU
SegFormer-B2	24.2M	0.851
SegFormer-B3	44M	0.848
SegFormer-B4	60.8M	0.856

**Table 4:** Evaluation metrics for ranking methods based on relative plant area and pod counting for indoor and outdoor datasets. Higher is better. Bold values denote best values achieved for each metric.

Location	Metric	Area (Mean)	Area (Med.)	Pod Points (Mean)	Pod Points (Med.)
Indoor	Kendall τ−β	0.869	0.816	**0.913**	0.860
	Spearman corr.	0.960	0.919	**0.972**	0.951
	NDCG@2	0.944	0.777	**0.964**	0.888
	NDCG@6	**0.969**	0.868	0.926	0.948
	NDCG@10	0.968	0.920	**0.987**	0.958
	NDCG@22	0.984	0.946	**0.989**	0.971
Outdoor	Kendall τ−β	0.736	0.807	**0.834**	0.745
	Spearman corr.	0.875	0.928	**0.942**	0.883
	NDCG@2	**0.964**	0.944	**0.964**	0.859
	NDCG@6	0.886	0.940	**0.961**	0.880
	NDCG@10	0.960	0.974	**0.983**	0.950
	NDCG@22	0.977	0.983	**0.986**	0.969

## Data Availability

The raw data supporting the conclusions of this article will be made available by the authors on request.

## Abbreviations

The following abbreviations are used in this manuscript:

AI	Artificial Intelligence
API	Application Programming Interface
CLIP	Contrastive Language Image Pretraining
CNN	Convolutional Neural Network
CPU	Central Processing Unit
CV	Computer Vision
CVAT	Computer Vision Annotation Tool
DETR	Detection Transformer
DINO	Distillation with NO labels
DINO detector	DETR with Improved Denoising Anchor Boxes
DL	Deep Learning
EMA	Exponential Moving Average
GPU	Graphics Processing Unit
GT	Ground Truth
IoU	Intersection Over Union
MAE	Mean Average Error
MCNN	Multi-Column Convolutional Neural Network
NMS	Non-Maximum Suppression
P2PNet	Point-to-Point Network
SAHI	Slicing-Aided Hyper-Inference
SAM	Segment Anything Model
VFM	Vision Foundational Model
ViT	Vision Transformer
YOLO	You Only Look Once
